# Intravital imaging of interactions between iNKT and kupffer cells to clear free lipids during steatohepatitis

**DOI:** 10.7150/thno.51369

**Published:** 2021-01-01

**Authors:** Haitao Wang, Longjun Li, Yinling Li, Yue Li, Yeqin Sha, Shuang Wen, Qiang You, Lixin Liu, Meiqing Shi, Hong Zhou

**Affiliations:** 1 Department of Immunology, Nanjing Medical University, Nanjing, Jiangsu 211166, China.; 2 Second Affiliated Hospital, Nanjing Medical University, Nanjing, Jiangsu 210011, China.; 3 Department of Anatomy, Physiology, and Pharmacology, College of Medicine, University of Saskatchewan, Saskatoon, Saskatchewan S7N 5E5, Canada.; 4 Division of Immunology, Virginia-Maryland College of Veterinary Medicine, University of Maryland, College Park, MD 20742, USA.

**Keywords:** iNKT cells, Kupffer cells, steatohepatitis, phagocytosis, intravital imaging

## Abstract

**Rationale:** Invariant natural killer T (iNKT) cells and Kupffer cells represent major hepatic populations of innate immune cells. However, their roles in steatohepatitis remain poorly understood. To elucidate their functions in steatohepatitis development, real-time, *in vivo* analysis is necessary to understand the pathophysiological events in the dynamic interactions between them during diet-induced steatohepatitis.

**Methods:** We used a steatohepatitis animal model induced by a methionine-choline-deficient (MCD) diet. Multi-photon confocal live imaging and conventional experimental techniques were employed to investigate the hepatic pathological microenvironment of iNKT and Kupffer cells, interactions between them, and the biological effects of these interactions in steatohepatitis.

**Results:** We found that iNKT cells were recruited and aggregated into small clusters and interacted dynamically with Kupffer cells in the early stage of steatohepatitis. Most significantly, the iNKT cells in the cluster cleared free lipids released by necrotic hepatocytes and presented a non-classical activation state with high IFN-γ expression. Furthermore, the Kupffer cells in the cell cluster were polarized to type M1. The transcriptome sequencing of iNKT cells showed upregulation of genes related to phagocytosis and lipid processing. Adoptive transfer of iNKT cells to Jα18^-/-^ mice showed that iNKT and Kupffer cell clusters were essential for balancing the liver and peripheral lipid levels and inhibiting liver fibrosis development.

**Conclusions:** Our study identified an essential role for dynamic interactions between iNKT cells and Kupffer cells in promoting lipid phagocytosis and clearance by iNKT cells during early liver steatohepatitis. Therefore, modulating iNKT cells is a potential therapeutic strategy for early steatohepatitis.

## Introduction

Steatohepatitis has gained increasing importance in Western countries. Although it has been considered a benign disease, *in vitro* studies have shown that persistent steatohepatitis develops into liver fibrosis and results in impaired insulin sensitivity and increased cardiovascular deaths 1, 2. Therefore, a better understanding of the associated immune cell functions is of great significance for preventing liver diseases and related systemic diseases. Although conventional experimental techniques help identify the molecular structure and functions of immune cell types in the liver, it is challenging to study the behavior, functional changes, and dynamic interactions between liver immune cells in their microenvironment. Therefore, we used a high-resolution real-time imaging technology, the multi-photon system, to image immune cell recruitment, observe the changes in liver cell dynamics, and study the occurrence and development of steatohepatitis.

Natural killer T (NKT) cells are a special subset of T lymphocytes expressing NK cell markers (e.g., NK1.1) and T cell receptors (TCR), depending on whether they are recognized by non-polymorphic MHC class I molecules and CD1d 3. NKT cells are divided into two types: type I NKT cells (iNKT) and type II NKT cells (vNKT) 4. Many studies have shown that iNKT cells are involved in the occurrence and development of liver diseases like drug-induced liver injury, hepatic fibrosis, hepatocellular carcinoma, and non-alcoholic fatty liver 5-9. There is an increased accumulation of iNKT cells in the liver in steatohepatitis compared to steatosis, indicating that the recruited iNKT cells play a key role in steatohepatitis development 10. A recent study showed that the differential activation of iNKT cells plays a key role in mediating diet-induced liver steatosis and fibrosis in mice 11. Earlier studies reported that fat-derived iNKT cells could improve insulin sensitivity and reduce body fat by producing IL-10 and had a potential involvement in human steatohepatitis 12, 13. These studies showed a direct or indirect regulation of lipid metabolism by iNKT cells through Th1/2 cytokines produced in different lipid microenvironments. However, the involvement of iNKT cells in lipid metabolism and the possible underlying mechanisms activity have not been investigated.

Kupffer cells are the resident macrophages of the liver. Numerous studies have implicated their essential role in the pathogenesis and progression of steatohepatitis 14, 15. Kupffer cell activation can lead to pro-inflammatory cytokine production, such as tumor necrosis factor (TNF)-α and IL-1β, critical mediators in steatohepatitis 16. Mechanistic studies in various steatohepatitis animal models also revealed that cholesterol crystals or saturated fatty acids activate and facilitate NOD-, LRR- and pyrin domain-containing protein 3 (NLRP3) complex assembly in Kupffer cells to further promote the maturation and release of pro-inflammatory cytokines 17-19. Additionally, a significant reduction in the liver fat content was reported following liposome-encapsulated clodronate injection in mice fed with methionine and choline-deficient feed (MCD feed) to remove Kupffer cells 20. Contrary to this observation, another study reported that liposome-encapsulated clodronate removed the liver Kupffer cells in mice, leading to a decrease in IL-10 secretion and promoted fat accumulation in the liver 21. Therefore, the Kupffer cell response to lipids in the pathogenesis of steatohepatitis is debatable.

Although significant progress has been made in identifying the key roles of iNKT and Kupffer cells in the fat metabolic diseases of the liver, little is known regarding the dynamic interactions between iNKT and Kupffer cells during the development of steatohepatitis. Therefore, to study the interactions between iNKT and Kupffer cells and the biological effects on the occurrence and development of steatohepatitis, we subjected Cxcr6*^Gfp/+^* transgenic mice to real-time resolution imaging, enabling visualization of the dynamic interaction between iNKT and Kupffer cells. Our results showed that the MCD feed led to a change in the normal movement pattern of iNKT cells, which interacted with Kupffer cells to aggregate and form clusters in an independent pathological microenvironment. Most importantly, we discovered that aggregated iNKT cells engulfed free lipids released by necrotic liver cells during early steatohepatitis. Furthermore, supplementation of iNKT cells reduced triglyceride levels in the liver and peripheral circulation while inhibiting hepatic fibrosis development.

## Methods

### Mice and steatohepatitis model

Male C57BL/6 mice were obtained from the Animal Model Center of Nanjing University (Nanjing, China). Cxcr6*^Gfp/+^* mice and Jα18^-/-^ mice were provided by Dr. Zhigang Tian (University of Science and Technology, School of Life Science, Hefei, China), and Cd1d^-/-^ mice were provided by Dr. Yongwen Chen (Army Medical University, Chongqing, China). Jα18^-/-^ and CD1d^-/-^ were backcrossed on the C57BL/6 background, both Jα18^-/-^ mice and Cd1d^-/-^ mice have normal immune systems and no pathological sensitivity unless they are attacked by specific pathogenic or tumorigenic factors. All mice (8 weeks old; 19-25 g) used for the experiments were housed in a pathogen-free, double-barrier unit at 22 °C in the Animal Research Centre of Nanjing Medical University under a 12-h light/dark cycle, with water and food provided ad libitum. In general, 8-wk-old male mice were fed a methionine-deficient diet to establish a non-alcoholic fatty liver disease model. For liver imaging studies, the mice received either MCS (Research Diets, A02082003B) or MCD (Research Diets, A02082002BR) from 8 weeks of age for 3, 5, or 7 days as indicated. According to the experimental protocol, more than 5 mice were in the control and experimental groups at different time points in various experiments. All protocols described herein were approved by the Nanjing Medical University Animal Care Committee (protocol #1802008) and were conducted in accordance with the guidelines established by the China Council on the Use of Laboratory Animals.

### Confocal laser scanning microscopy

Before intravital imaging, mice were anesthetized by intraperitoneal injection with ketamine and xylazine (100 and 10 mg/kg, respectively). Preparation of the murine liver for intravital microscopy was described previously 22-24. Intravital microscopy was performed with an LSM880 multi-photon-photon laser-scanning microscope (Zeiss, Oberkochen, Germany) equipped with an Olympus focus drive and motorized stage (Applied Scientific Instrumentation, Eugene, OR, USA). Visualization of iNKT cells and other immune cells in the liver vasculature was achieved with five laser-excitation wavelengths in rapid succession (405nm, 488 nm, 561 nm, 594 nm, and 633 nm) and captured with appropriate band-pass filters (Semrock and Chroma). For each experiment, a minimum of three fields were imaged per mouse over 2 h. Cell movements were analyzed using Imaris 7.4.2 (Bitplane, Zurich, Switzerland).

### Animal treatment

Transgenic Cxcr6*^Gfp/+^* mice were used to visualize hepatic iNKT cells 22. iNKT cells were labeled with 10 μg APC-conjugated CD1d-α-Galcer tetramers antibodies to verify that GFP cells were iNKT cells. Natural Killer (NK) cells were depleted by injection of 10 μg of purified anti-GM1. Kupffer cells were labeled intravenously with 10 μg Alexa Fluor 647-conjugated F4/80 antibodies (BioLegend, San Diego, CA, USA). Kupffer cell depletion was achieved by injecting 200 μL clodronate liposomes (CLL) (Yeasen Biotechnology, Shanghai, China) into the tail vein 24 h before MCD diet-induced non-alcoholic steatohepatitis (NASH). Lipid droplets were labeled by injecting 5 μg of the fluorescent probe Nile red (MedChemExpress, Monmouth Junction, NJ, USA). Debris resulting from oxidative stress was labeled by injecting 20 μg of the fluorescent ROS probe dihydroethidium (DHE; Vigorous Biotechnology, Beijing, China) into the tail vein of each mouse. ROS blockade was performed by injecting mice intravenously with 10 μg uric acid (UA; CSNpharm, CSN20795, USA) 12 h before MCD treatment. Liver sinusoids were visualized by injecting 10 μg Alexa Fluor 647-conjugated platelet endothelial cell adhesion molecule (PECAM)-1 antibodies (BioLegend). CD1d molecular localization was visualized by injecting 10 μg Alexa Fluor 647-conjugated CD1d molecule antibodies (BioLegend). CD86 molecular localization was visualized by injecting 10 μg PE-conjugated CD86 molecule antibodies (BioLegend). To verify the function of the Fcgr4 (CD16.2) gene detected by transcriptome screening, it was blocked by intravenous injection of 200 μg of purified anti-CD16.2 antibodies or 200 μg IgG isotype control 12 h before MCD treatment. To study the effects of α-galactosylceramide (α-GalCer)-based activation on iNKT cells, 5 μg α-GalCer (BioVision, Milpitas, CA, USA) was injected into the tail vein of each mouse before imaging mouse liver iNKT cell migration 22-24.

### Flow cytometry

Liver tissue was gently homogenized by grinding and mincing. Hepatic lymphocytes were purified by Percoll (Pharmacia, New York, NY, USA) gradient centrifugation. In GFP cells, iNKT cell populations were characterized by staining with allophycocyanin (APC)-conjugated anti-CD1d-α-Galcer tetramers (BioLegend) for 30 min at 4 °C. Dead cells were excluded from the analysis by adding 1 μL of Zombie NIR (APC-Cy7; BioLegend) 10 min prior to flow cytometry. The number of NKT cells in each liver sample was calculated by flow cytometry. The frequency of iNKT cells expressing PE/CY7-CD69 (BioLegend) and PE-CD25 (BioLegend) was determined by cell membrane staining. The frequency of iNKT cells expressing Percp/Cyanine5.5 IFN-γ (BioLegend) and PE-IL-4 (BioLegend) was analyzed by intracellular staining. Phenotypic analysis was performed using the FACS Calibur Cell Analyzer (Becton Dickinson, Franklin Lakes, NJ, USA) and FlowJo software (version 10.0.7; NIH, Bethesda, MD, USA) for data analysis.

### Liver tissue clearing

Cxcr6*^GFP/+^* mice were anesthetized with a mixture of ketamine and xylazine and perfused with 4% paraformaldehyde to obtain fresh liver tissue, as previously described 25. To prevent attenuation of the GFP signal, the liver tissue clearing procedure was completed quickly. The liver tissues were spliced into slices with a thickness <2 mm under a stereoscopic dissecting microscope. Liver tissue degreasing and transparent treatment were performed following the instructions of PEGASOS (China, PSK 100P/1950150054). Due to the thinness of the liver tissue, the entire clearing process was completed in 4-5 hours. The liver tissue has been cleared and labeled with APC-IFN-γ for multi-photon microscopy.

### Visualizing collagen by multi-photon microscopy

The second harmonic microimaging technique was used to detect positive signal generated by the non-linear characteristics of tissue collagen, as previously described 26. The excised mouse liver was stored in cold PBS and imaged with a FV1000MPE Olympus multi-photon microscope equipped with a sapphire laser. Using an excitation wavelength of 820 nm, the multi-photon fluorescence and second harmonic signals were used to visualize the liver tissue. An appropriate band-pass filter (Rhodamine, 580-610 nm; SHS, 405-415 nm) and non-descanned detectors were used on a vertical two-photon excitation microscope (TPEM) system (A1RMP, Nikon, Tokyo, Japan). Image acquisition was performed on the second harmonic signal generated by the collagen in the liver.

### Adoptive transfer of iNKT cells into Ja18^-/-^ mice

Cxcr6*^Gfp/+^* mice were injected with 200 μg of anti-asialo GM1 antibody via the tail vein to eliminate NK cells, as previously described 12, 22. After 24 h, the non-parenchymal cells were isolated from the liver and spleen of Cxcr6*^Gfp/+^* mice. Using flow cytometry, the GFP^+^CD1d-α-GalCer^+^ cells were collected as iNKT cells and re-suspended in sterile saline at a concentration of 5 × 10^6^ cells/ml. The recipient Jα-18^-/-^ mice were anesthetized with ketamine and xylazine, and 200 μL of the cell suspension was injected into the hepatic portal vein at a rate of 1 ml/min.

### Video microscopy and statistical analysis

Intravital single-photon laser scanning microscopy (SPLSM) data were analyzed using Imaris 7.4.2 (Bitplane). Cells were tracked based on their fluorescent properties using Imaris Cell, Imaris Track, and Imaris Measurement Pro. Cells and molecules were identified based on their fluorescent properties using Imaris Surfaces, and movies produced using Imaris Video.

We used the tracking function of Image J software and the Chemotaxis and Migration Tool to draw a discrete map of cell motion trajectory. Cellular motility was determined by MATLAB (MathWorks) software measuring speed, displacement, arrested coefficient, confinement ratio, and mean displacement (μm) versus the square root of the time (min^1/2^). All data were expressed as means ± SEM. Data were compared using an unpaired two-tailed Student's *t*-test or one-way analysis of variance (ANOVA) with GraphPad Prism software (version 5.02; GraphPad Software Inc., La Jolla, CA, USA). Bonferroni's multiple comparisons test was performed with SPSS (version 17.0 for Windows; IBM, Armonk, NY, USA).

Additional methodology details are provided in the supplementary material.

## Results

### Intravital imaging of the liver shows dynamic changes in the iNKT cell recruitment and clustering in the liver

The MCD diet produced significant hepatic steatosis, as evidenced by markedly increased Oil Red O positive staining in the liver (Figure S1A). Compared with methionine- and choline-supplemented (MCS) diet-fed mice, those fed an MCD diet for 3 and 5 days, respectively, exhibited significantly higher levels of hepatic TGs (Figure S1B) and aminotransferases (AST and ALT; Figure S1C). We also found that mRNA levels of inflammatory cytokines IL-6, TNF-α, and IL-1β were significantly increased in liver tissues (Figure S1D). Although induced by MCD for a short time, the steatohepatitis characteristics were evident in mice livers.

**Figure 1:**
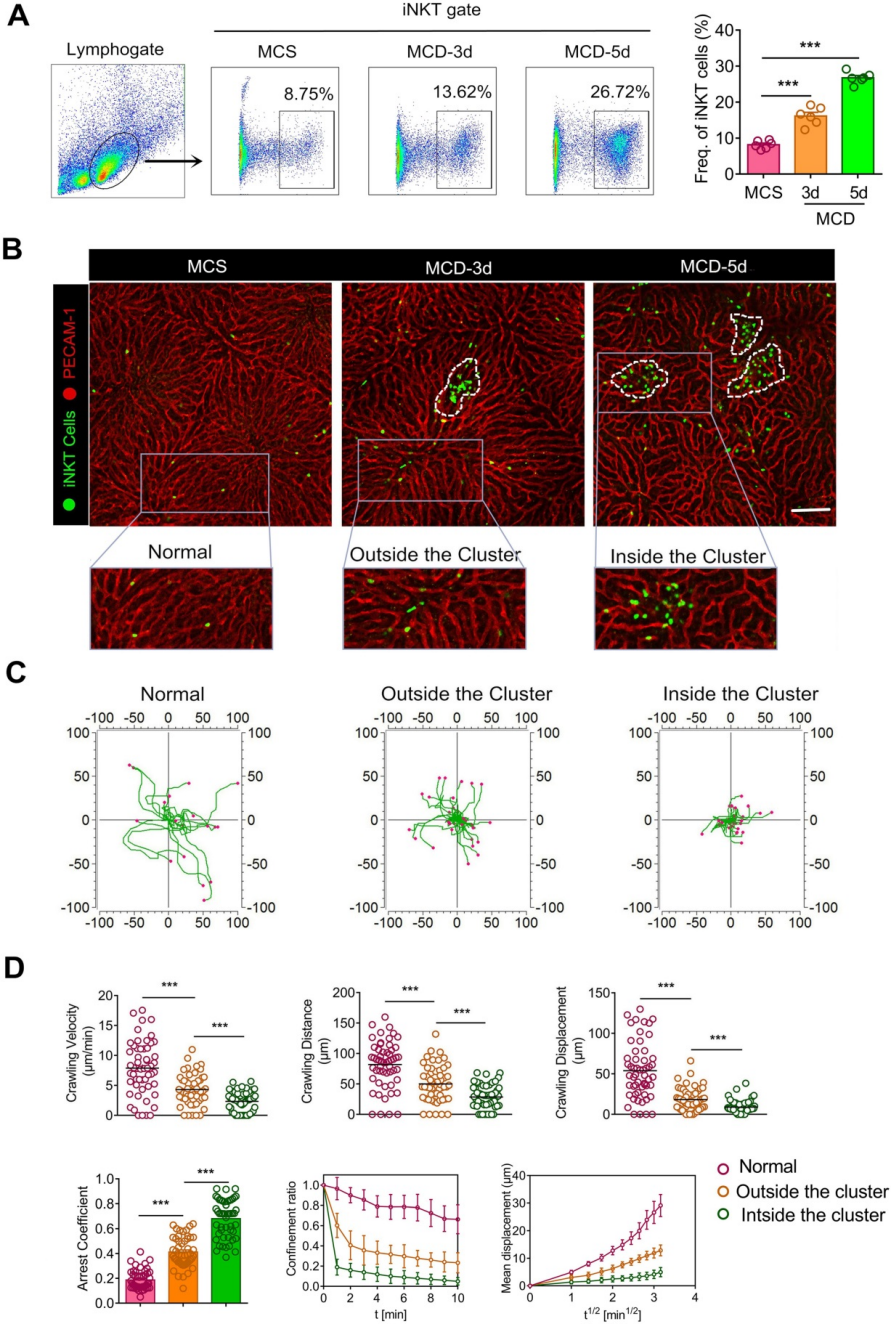
** Recruited iNKT cells form aggregated clusters and display distinct migratory patterns in early** steatohepatitis**. (A)** Left: Frequencies of iNKT cells (GFP^+^CD1d^-^tetramers^+^) among liver leukocytes of C57BL/6 mice (n = 6) fed MCS or MCD diet for 3 or 5 days, as evaluated by flow cytometry. Right: Quantification of recruited iNKT cells in mice livers fed MCS or MCD diet for 3 or 5 days (n = 6). **(B)** Cxcr6*^Gfp/+^* mice fed MCS or MCD diet for 3 or 5 days were subjected to hepatic intravital microscopy. Representative images were obtained from ≥ 3 independent experiments to examine iNKT cell response (green). The sinusoidal endothelium was labeled using Alexa Fluor 647-conjugated anti-platelet endothelial cell adhesion molecule (PECAM-1) antibodies (red). The image in the square with a gray outline is partially enlarged. iNKT cells were divided into three states: normal state, outside the cluster, and inside the cluster. Scale bar, 100 µm. iNKT cell clusters are indicated with a white dashed line. **(C)** Overlay of three states iNKT cell migratory tracks plotted after aligning their starting positions. Each plot was collected from 20 iNKT cells from three different Cxcr6*^Gfp/+^* mice, and each scan was imaged for 10 min. **(D)** Scatter plots of the velocity, distance, and migratory displacement of migrating iNKT cells in the liver of Cxcr6*^Gfp/+^*mice (n = 3) fed MCS or MCD diet for 3 days. Scatter plots or displacement curves of the arrested coefficient, Confinement ratio, and mean displacement (µm) versus the square root of the time (min^1/2^) of migrating iNKT cells in the liver of Cxcr6*^Gfp/+^* mice (n = 3) fed an MCS or MCD diet for 3 days. Fifty migrating iNKT cells from three independent experiments were pooled. Data represent mean relative expression ± SEM. *P < 0.05; **P < 0.01; ***P < 0.001 using two-tailed unpaired student's t-test.

To study the recruitment and dynamics of iNKT cells in steatohepatitis, we employed Cxcr6*^Gfp/+^*transgenic mice, widely used to study the behaviors and functions of iNKT cells in living tissues 22, 27, 28. Flow cytometry of hepatic leukocyte populations revealed that the MCD diet considerably enhanced iNKT cell recruitment into the liver after 3 and 5 days (Figure 1A). The proportion of recruited GFP cells and CD1d-α-Galcer positive cells was the same and was classified as the same gate, indicating the identity of recruited GFP cells as iNKT cells. The time for MCD-induced steatohepatitis in our study was shorter than previous studies 14, 18; however, iNKT cell recruitment was markedly increased in NASH.

Next, we performed intravital liver imaging using a high-resolution confocal microscope. We observed that iNKT cells were uniformly distributed and randomly patrolled in the liver sinusoid of Cxcr6*^Gfp/+^* mice deprived of MCD treatment. However, the migratory trajectories of iNKT cells became more limited with the MCD diet (Figure 1B, Video S1). Importantly, we also noticed that the recruited iNKT cells exhibited clustered and non-clustered states in the liver (Figure 1B). Enhanced iNKT cell recruitment was observed on the third day, with the number of recruited iNKT cell clusters increasing over time (Figure S2A). iNKT cells in the liver exercised certain displacements outside the cluster, while those within the cluster showed minimal displacement and a characteristic restricted movement (Video S2). We also used CD1d-α-Galcer to further identify GFP iNKT cells in cell clusters and eliminate the cross-talk from other cells, like NK cells expressing the Cxcr6 receptor (Figure S3 A). Additionally, we used Imaris and Image J software to measure the iNKT cell dynamics in different states and depict a discrete cell migration strategy (the actual starting point of each cell movement being the coordinate center). Our findings clearly demonstrated characteristic changes in iNKT cell kinetics in exercising restriction during steatohepatitis (Figure 1C). We used quantitative statistics to show the difference in iNKT cell (Fifty cells) dynamics in three different states digitally, as previously described 29. Compared to the other two states, iNKT cells within the cluster showed minimal rate of movement, distance moved, displacement, confinement ratio, and mean displacement (μm) versus the square root of time (min^1/2^) and maximum arrested coefficient (Figure 1D). These data show that iNKT cells within the cluster exhibited a significant restricted migratory characteristic in steatohepatitis.

Endothelial barrier dysfunction of microcirculation mainly manifests in vascular endothelial cell damage 30. We injected 10 μg of PECAM-1 into the tail vein of mice to label blood vessels and evaluated the fluorescence intensity of blood vessels in iNKT cell clusters. We found that the fluorescence continuity of endothelial cells in the iNKT cell cluster was interrupted or the fluorescence blur disappeared (Figure S3B), indicating damage to endothelial cells in the iNKT cell cluster and impairment of the endothelial barrier function. We also found that the CXCR6 receptor ligand, CXCL16 increased significantly after 36 hours of MCD induction (Figure S3C). The data showed that iNKT cells were recruited and clustered in the liver during steatohepatitis development. Also, the iNKT cell dynamic was altered and the endothelial barrier function impaired in the microenvironment of iNKT cell aggregation sites.

### iNKT cell clusters exhibit an activation pattern different from the classical α-GalCer stimulation

**Figure 2:**
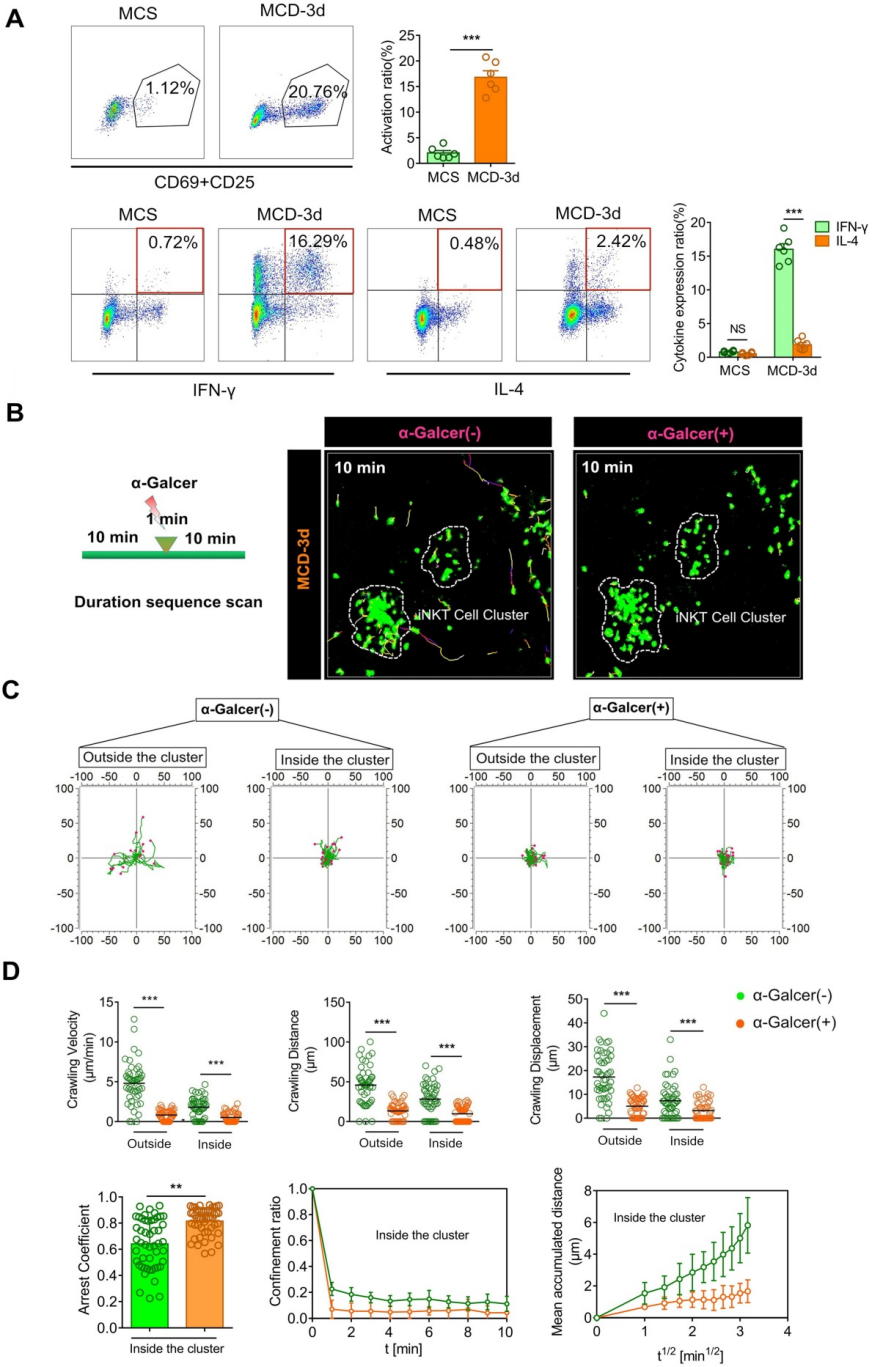
** iNKT cell clusters exhibit an activation pattern different from the classical α-GalCer stimulation. (A)** Top: Representative flow cytometry image from three experiments of CD69 and CD25 expression of iNKT cells (GFP + CD1d-tetramer) obtained from Cxcr6*^Gfp/+^*mice (n = 6) fed MCS or MCD diet. Bottom: Representative flow cytometry image from three experiments of intracellular IFN-γ or IL-4 staining of iNKT cells (GFP + CD1d-tetramer) in Cxcr6*^Gfp/+^* mice (n = 6) fed MCS or MCD diet. **(B)** Representative images showing the migratory paths of iNKT cells in Cxcr6*^Gfp/+^* mice (n = 5) before and after treatment with α-GalCer. Each area was imaged for 10 min. Scale bar, 100 µm. **(C)** Alignment of the starting position of the iNKT cell migratory trajectory, covered before and after iNKT cell activation. Data were collected from three mice, and each scan was imaged for 10 mins. **(D)** Velocities, distance, and displacement of migrating iNKT cells in Cxcr6*^Gfp/+^* mice (n = 3), before and after α-GalCer treatment. Scatter plots or displacement curves of the arrested coefficient, confinement ratio, and mean displacement (µm) versus the square root of the time (min^1/2^) of migrating iNKT cells in the iNKT clusters before and after α-GalCer treatment. Fifty iNKT cells from 3 independent experiments were pooled. Data represent mean relative expression ± SEM. *P < 0.05; **P < 0.01; ***P < 0.001 using two-tailed unpaired student's t-test.

Previous studies have shown elevated IL-4 and IFN-γ mRNA expression in stationary phase iNKT cells 31, 32. It has been found that, upon stimulation by α-GalCer, iNKT cells were rapidly activated and produced large amounts of IL-4 and IFN-γ 33. CD69 and CD25 levels, characteristics of iNKT cell activation, were significantly increased upon stimulation by the MCD diet, as observed by flow cytometry analysis (Figure 2A, Up). There was a sharp increase in the IFN-γ expression level of iNKT cells, while the increase in IL-4 expression level was not significant (Figure 2A, Down). We also observed a higher expression level of IFN-γ in iNKT cell clusters by three-dimensional imaging (Figure S4A).

Previous studies have shown that normally-moving iNKT cells in the liver quickly stop moving when stimulated by α-GalCer, a specific iNKT cell activator 34. Therefore, we injected mice with α-GalCer to examine its effect on the behavior of iNKT cells in the cluster. Within one minute of injecting α-GalCer into the tail vein, the iNKT cells that were freely crawling outside the cluster began exhibiting limited motility and subsequently became static. Likewise, the cell motility of iNKT cells in the cell cluster also stopped following α-GalCer injection, which was significantly different from that before α-GalCer stimulation. Also, scatter plots or displacement curves of the arrested coefficient, confinement ratio, and mean displacement (μm) versus the square root of the time (min^1/2^) of iNKT cell migration in the cell clusters showed different movement states before and after α-GalCer treatment. Thus, α-GalCer eliminated the dynamic difference between iNKT cells outside and inside the cluster and normalized the cell dynamics (Figure 2B-D, Video S3). These results indicated that during steatohepatitis, the iNKT cells in the cluster exhibited an activated phenotype and different cell dynamics patterns compared to iNKT cells classically stimulated by α-GalCer.

### Visualization of hepatocellular necrosis in iNKT cell clusters

**Figure 3:**
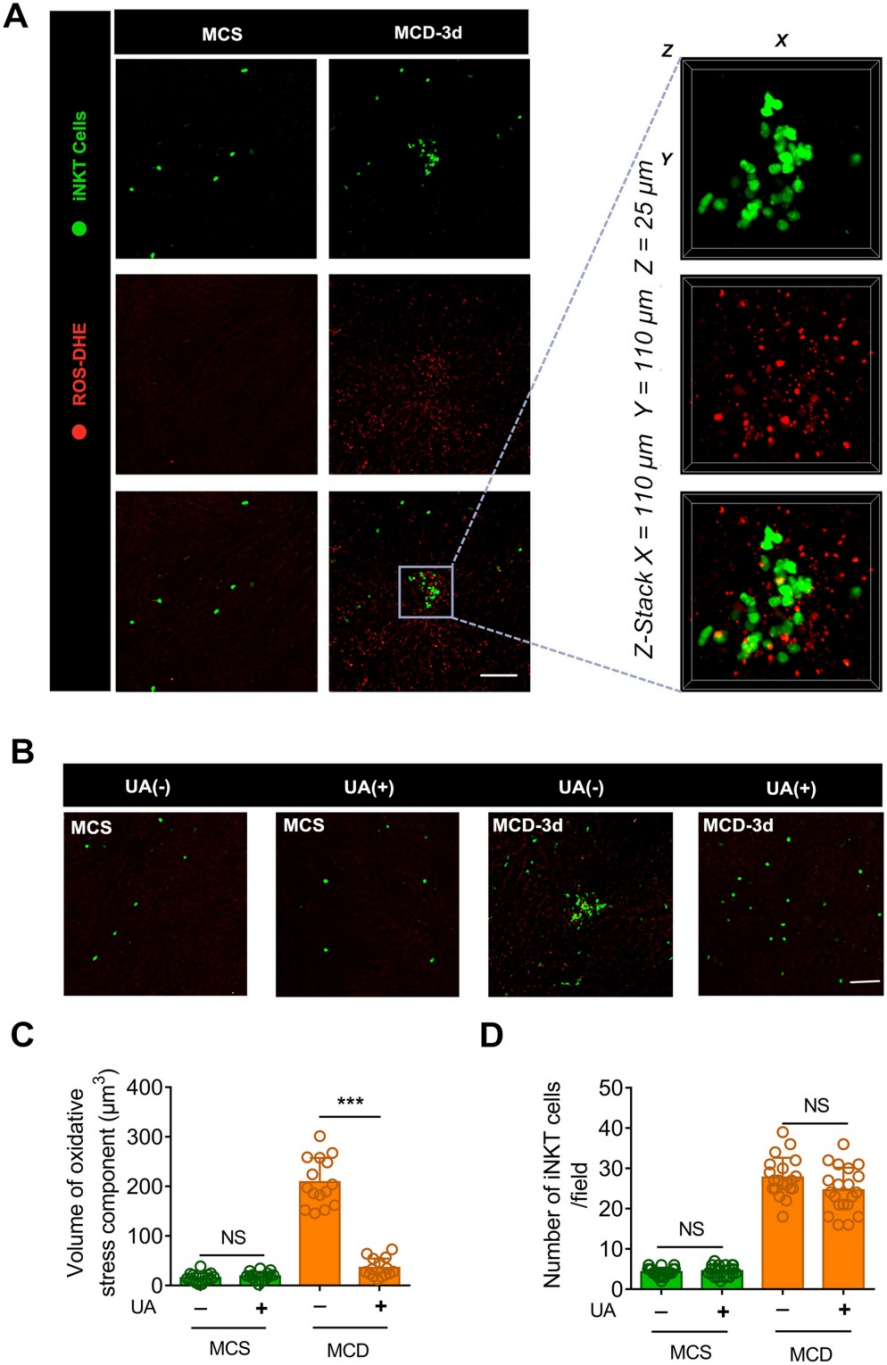
** Uric acid (UA) blocks the aggregation of iNKT cells in necrotic inflammatory lesion areas. (A)** Representative images from ≥ 3 independent experiments in Cxcr6*^Gfp/+^*mice fed MCS or MCD diet for 3 days. Reactive oxygen species (ROS) were labeled using the fluorescent probe dihydroethidium (DHE, red). The image in the square with a gray outline is partially enlarged. Representative 3D images showing DHE-labeled ROS in an iNKT cell cluster (green). Three-dimensional image dimensions, X = 110 µm, Y = 110 µm, Z = 25 µm, where the XY plane is perpendicular to the objective lens. **(B)** Representative images from ≥ 3 independent experiments to examine the number of recruited iNKT cells (green) in the liver of mice fed MCS or MCD diet for 3 days, with or without NAC treatment. ROS were labeled with DHE (red), Scale bar, 100 µm.** (C)** Quantification of the volume of oxidative stress debris in Cxcr6*^Gfp/+^*mice (n = 5) treated with or without UA. **(D)** Quantification of the number of liver iNKT cells in Cxcr6*^Gfp/+^* mice (n = 5) treated with or without UA. Data represent mean relative expression ± SEM. *P < 0.05; **P < 0.01; ***P < 0.001 using two-tailed unpaired student's t-test.

Previous studies have shown that excessive lipid droplet accumulation induces oxidative stress in the hepatocyte mitochondria and endoplasmic reticulum during the early stages of steatohepatitis, ultimately resulting in hepatocyte necrosis 35. We injected mice with the fluorescent reactive oxygen species (ROS) probe DHE and Hoechst 33342 to label the oxidative components and nuclei in the liver. We then performed intravital microscopy to investigate the oxidative microenvironment of aggregated iNKT cell clusters. A minimal level of oxidative stress was observed in the livers of MCS diet-fed mice (Figure 2A). Conversely, fluorescence due to ROS staining was abundant in the aggregated iNKT cell clusters in mice fed an MCD diet (Figure 3A, Video S4). Three-dimensional (3D) analysis further revealed that numerous DHE-labeled ROS fragments were detected in the aggregated iNKT cell clusters (Figure 3A, Video S5). Therefore, we speculated that the iNKT cell cluster formation was intrinsically associated with hepatocyte necrosis.

To confirm our hypothesis, we injected uric acid (UA) in the tail vein of mice fed with MCD. UA is an endogenous antioxidant that can scavenge reactive ROS, including singlet oxygen, oxygen-free radicals, and peroxynitrite 36, 37. Uric acid significantly reduced ROS levels and blocked iNKT cell clustering in the liver of MCD diet-fed mice (Figures 3B-C). This occurred without affecting the number of recruited iNKT cells in the liver (Figure 3D). Furthermore, histopathology staining showed diffuse vesicular steatosis and local hepatocyte necrosis in the MCD-induced liver tissue. However, there was no obvious hepatocyte necrosis in the liver tissue of the uric acid intervention group (Figure S5A). Therefore, a pathological microenvironment rich in chemokines, lipids, or ROS-inducing substances released by damaged endothelial cells or necrotic hepatocytes might contribute to recruited iNKT cell aggregation.

### Kupffer and iNKT cells cluster together and interact dynamically

**Figure 4:**
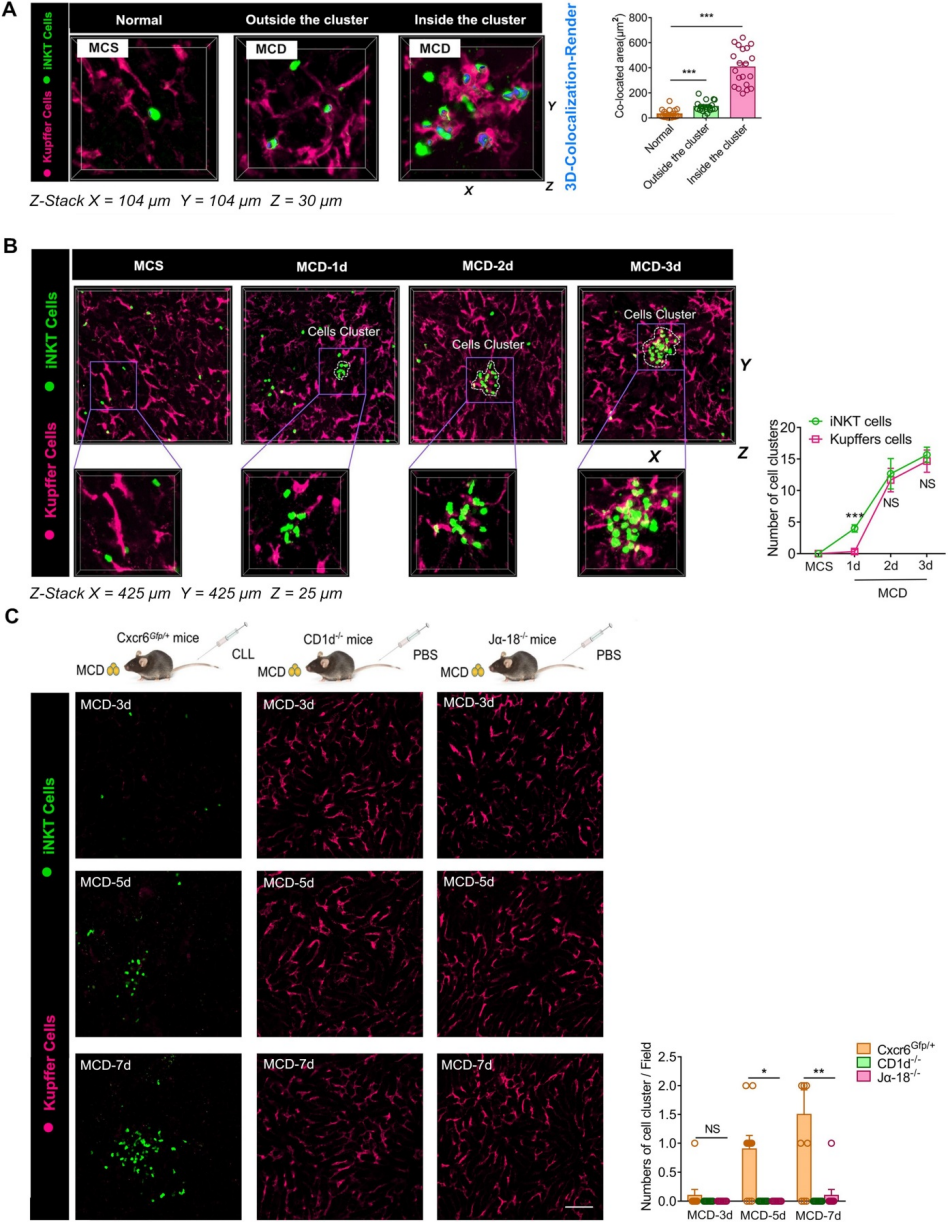
** Kupffer and iNKT cells cluster together and interact dynamically, and aggregation properties of Kupffer cells depend on the aggregation of iNKT cells. (A)** Left: Representative Z-Stack fluorescence co-localization images (X = 100 µm, Y = 100 µm, Z = 25 µm) were obtained from ≥ 3 experiments performed to examine the interactions between iNKT cells and Kupffer cells in different states. Three-dimensionally rendered blue was used to indicate iNKT and green for Kupffer cell (Fuchsia, labeled with Alexa Fluor® 647-conjugated anti-mouse F4/80 antibodies) interactions. Right: Quantification of the co-located area of iNKT and Kupffer cell interactions in three states (n = 20). **(B)** Left: Cxcr6*^Gfp/+^* mice fed MCS or MCD diet were subjected to hepatic intravital microscopy. Representative Z-Stack images (X = 425 µm, Y = 425 µm, Z = 25 µm) were obtained from ≥ 3 experiments performed to examine the interactions between iNKT cells (green) and Kupffer cells (Fuchsia, labeled with Alexa Fluor® 647-conjugated anti-mouse F4/80 antibodies). Right: Quantification of the number of iNKT cell clusters and Kupffer cell clusters in untreated or MCD-fed Cxcr6*^Gfp/+^* mice (n = 5). **(C)** Left: Representative images were obtained from 3 independent experiments to examine the formation ability of cell clusters composed of iNKT cells (green)and Kupffer cells (Fuchsia, labeled with Alexa Fluor® 647-conjugated anti-mouse F4/80 antibodies). WT (Cxcr6*^Gfp/+^*mice) or iNKT cell-deficient mice (CD1d*^-/-^*mice, Jα18*^-/-^*mice) treated with clodronate liposomes (CLL) or PBS fed MCD diet for 3, 5, and 7 days. Scale bar, 100 µm. Right: Quantification of the number of iNKT cell clusters and Kupffer cell clusters in WT (Cxcr6*^Gfp/+^*mice) or iNKT cell-deficient mice (CD1d*^-/-^*mice, Jα18*^-/-^*mice) (n = 5). Data represent mean relative expression ± SEM. *P < 0.05; **P < 0.01; ***P < 0.001 using two-tailed unpaired student's t-test.

As a potential antigen-presenting cell, the Kupffer cell is involved in the T lymphocyte activation in the liver 38, 39. We tested if Kupffer cells interacted with iNKT cells in the cluster. We found that Kupffer cells in iNKT cell clusters also formed clusters, and these aggregated Kupffer cells interacted dynamically with iNKT cells recruited to the cluster (Video S6). We identified clustering together of iNKT and Kupffer cells by three-dimensional imaging (Video S7). We performed a co-localization analysis of the contact surfaces and found that the two cell types displayed significant interactions in the cell cluster (Figure 4A). Since the phenotype and functioning of Kupffer cells play an important role in the development of various acute and chronic liver diseases 40, 41, we further analyzed the possible phenotypes of the aggregated Kupffer cells and found high expression levels of CD86, which is a characteristic feature of M1 polarization (Figure S6A). We also found a high expression of CD1d in aggregated Kupffer cells (Figure S6B).

Although we visually monitored the aggregation of iNKT and Kupffer cells in the necrotic hepatocytes of mice fed with MCD for 3 days, the order of iNKT and Kupffer cell cluster formation was not clear. Therefore, we modified the time interval for monitoring mice following MCD feed. We detected recruitment and slight aggregation of iNKT cells in the liver of mice on the first day following the MCD feed consumption, but the Kupffer cells did not aggregate at this time (Figure 4B). From the second day onwards, Kupffer cells also began aggregating slightly along with the iNKT cells (Figure 4B). We speculated that iNKT and Kupffer cells might have interdependent characteristics. Therefore, we used clodronate liposomes (CLL) to remove macrophages from CXCR6*^Gfp/+^* mice to observe iNKT cell aggregation, and CD1d*^-/-^*and Jα18*^-/-^* mice of the same age to observe Kupffer cell aggregation. In the absence of Kupffer cells, iNKT cells were no longer recruited and aggregated at the initial stage, but after day 5 of MCD induction, iNKT cells were recruited and aggregated (Figure 4C). However, in iNKT-deficient mice (CD1d^-/-^ and Jα18^-/-^), no significant aggregation of Kupffer cells was observed with MCD induction (Figure 4C). These observations indicated that Kupffer cells could regulate the early recruitment of iNKT cells, but the depletion of Kupffer cells did not prevent iNKT cell recruitment and aggregation for a long time. We also did not find a significant difference in the iNKT cells' virulence in different states on primary liver cells (Figure S7A).

Finally, we compared the changes in TH1/2 cytokine (TH1: IL-1β, TNF-α; TH2: IL-5, IL-10) mRNA levels in iNKT and Kuffer cells under MCS or MCD-induced conditions and found that Kupffer cells were more likely to enhance pro-inflammatory response, while iNKT cells inhibited the inflammatory response (Figure S7B). The data indicated that both iNKT and Kupffer cells formed clusters by MCD induction, and the aggregation characteristics of Kupffer cells appeared to be more dependent on the presence of iNKT cell clusters. These findings revealed a significant time and space-dependent interaction pattern in the aggregated clusters of iNKT and Kupffer cells.

### Intravital imaging reveals phagocytosis of lipids by iNKT cells in cell clusters

**Figure 5:**
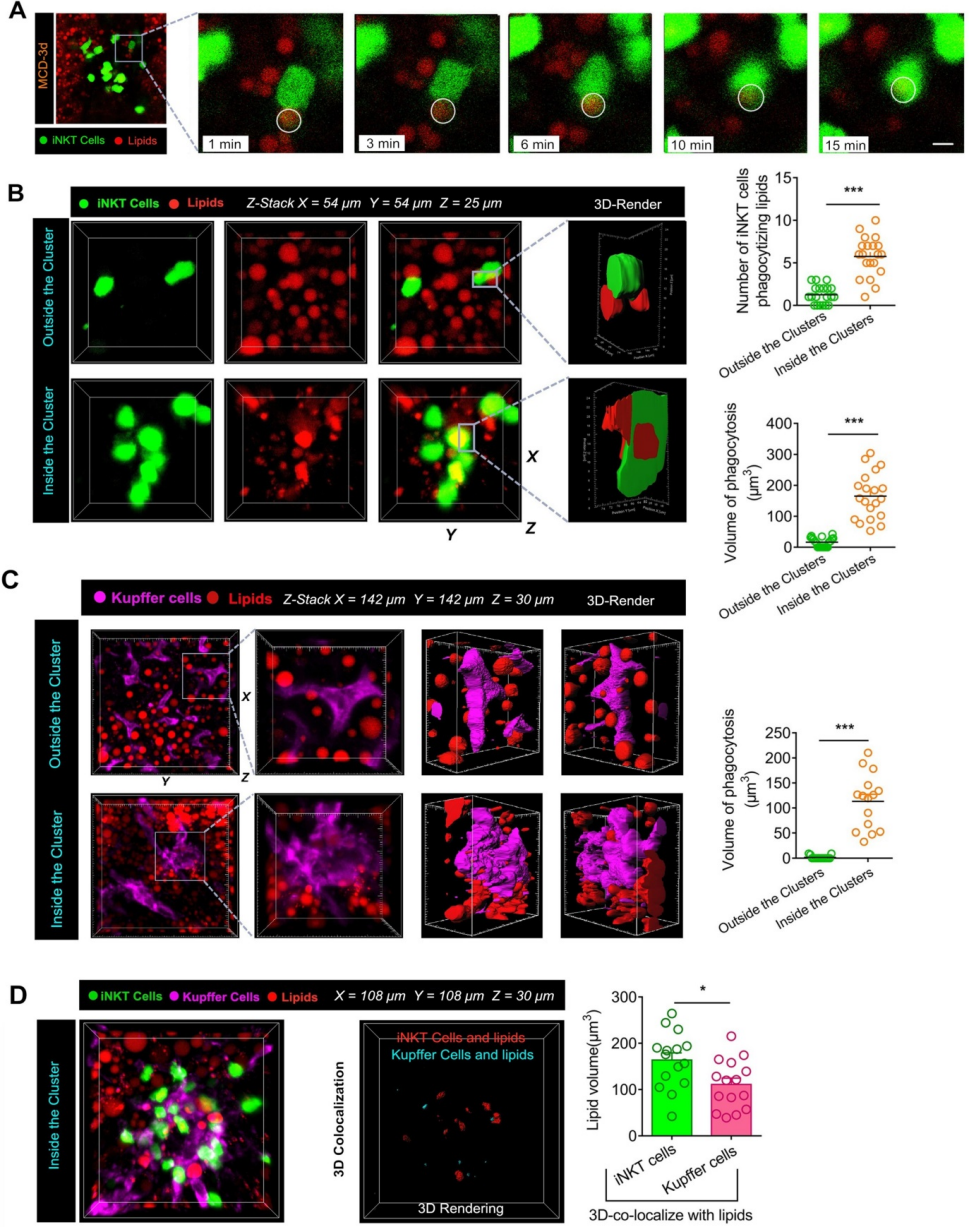
** Aggregated iNKT cells have the lipid phagocytosis ability. (A)** Cxcr6*^Gfp/+^* mice fed MCD diet for 3 days were subjected to hepatic intravital microscopy. Time-lapse images show snapshots of a green iNKT cell (green) approaching a triglyceride (TG) drop (stained with Nile red and indicated with a white circle) and eventually engulfing it. See also Supplementary Video 8. **(B)** Left: Representative 3D images from ≥ 3 independent experiments showing aggregated iNKT cells (green) and TG drops (red, stained with Nile red) in the liver of Cxcr6*^Gfp/+^* mice fed MCD diet. 3D image dimensions (µm): X = 54 µm, Y = 54 µm, Z = 25 µm, where the XY plane is perpendicular to the objective lens. Representative 3D intersection images and single-cell cutting (after 3D surface rendering) show the hepatic iNKT cell (green) ability to engulf lipid drops (red). Right: Quantification of iNKT cell number with TG drops and volume of lipid drops engulfed in the cytoplasm outside or inside the iNKT cell cluster in the liver (n = 20). **(C)** Left: Representative images were obtained from ≥ 3 experiments to examine lipids (red, Nile red labeled lipid) in clustered or non-aggregated Kupffer cells (purple, F4/80 antibody-labeled Kupffer cells). 3D images of Kupffer cells and lipids obtained by Z-stacking were scanned. Kupffer cells (3D rendering) in the cell cluster show co-localization with the lipid. X = 142 µm, Y = 142 µm, Z = 30 µm, where the XY plane is perpendicular to the objective lens. Right: Quantification of volume of lipid drops engulfed by Kupffer cells outside or inside the clusters in the liver (n = 15). **(D)** Left: Representative images were obtained from ≥ 3 experiments to examine lipids (red, Nile red labeled lipid) in iNKT cells (green) and Kupffer cells (purple, F4/80 antibody-labeled Kupffer cells). 3D images of iNKT cells, Kupffer cells, and lipids obtained by Z-stacking were scanned. iNKT cells (co-localized in red, 3D rendering) and Kupffer cells (colocalized in aqua blue, 3D rendering) in the cell cluster show co-localization with the lipid. X = 108 µm, Y = 108 µm, Z = 30 µm, where the XY plane is perpendicular to the objective lens. Right: Quantification of the volume of engulfed lipid drops in aggregated iNKT cells or Kupffer cells in Cxcr6*^Gfp/+^* mice fed MCD diet (n = 15). Data represent mean relative expression ± SEM. *P < 0.05; **P < 0.01; ***P < 0.001 using two-tailed unpaired student's t-test.

The inflammatory microenvironment of iNKT and Kupffer cell clusters contains numerous cell components, which are released following the necrosis of hepatocytes. During steatohepatitis, the components released by necrotic hepatocytes mostly consists of triglycerides rich in free fatty acids. We injected 5 μg of Nile Red lipid dye into the tail vein of mice to label triglycerides in the liver prior to intravital imaging. When induced with MCD, most iNKT cells in the cell clusters were restricted in movement, and the free triglycerides released by liver cell necrosis became flexible under the agitation of iNKT cells. Next, we captured a novel phenomenon. When the iNKT cells in the clusters were exposed to free lipids for about 15 minutes, the free triglycerides were completely engulfed by the iNKT cells. This dynamic process of iNKT cells phagocytosing free lipids was captured and retained by our high-resolution live imaging (Figure 5A, Video S8). Furthermore, a 3D reconstruction showed that a large proportion of iNKT cells had either triglycerides attached to their surface or engulfed lipid drops inside their cell bodies (Video S9). Interestingly, most of these lipid-engulfing iNKT cells were found in areas of aggregated iNKT clusters, whereas free-crawling iNKT cells outside the aggregated iNKT cell clusters engulfed lipids to a much less extent (Figure 5B). Consequently, the total volume of lipids engulfed in the aggregated iNKT cells was significantly higher than in freely moving iNKT cells outside the clusters (Figure 5B).

We found that co-localization of Kupffer cells and lipid droplets occurred in clusters only in mice consuming the MCD diet. In a three-dimensional analysis of the interaction between Kupffer cells and lipids in the livers of mice fed MCD diet, direct contact between Kupffer cells and lipid contents was only noted in aggregated cell clusters (Figure 5C). Additionally, we performed a 3D quantitative analysis of lipids in iNKT and Kupffer cell clusters. Results showed that the co-localization between iNKT cells and lipids was significantly higher than in Kupffer cells and lipids (Figure 5D). These results indicated that the cells phagocytosing lipids in the cell cluster were mainly iNKT cells.

### Recruited iNKT cells display unique mRNA expression patterns associated with lipid processing

**Figure 6:**
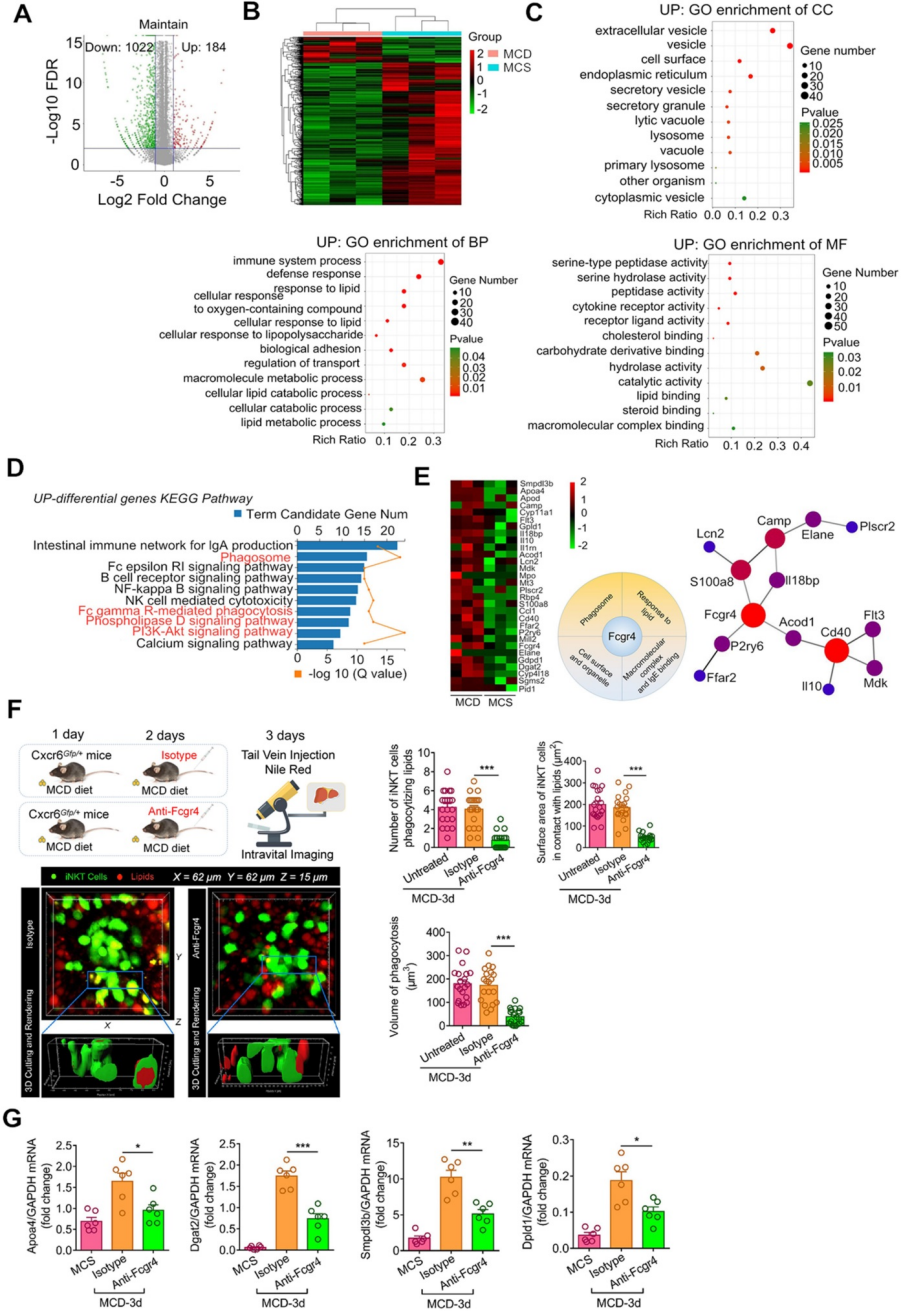
** The recruited iNKT cells display unique mRNA expression patterns associated with lipid processing. (A)** Differentially expressed genes and their levels in iNKT cells from MCD diet-fed Cxcr6*^Gfp/+^* mice are displayed in volcano maps and a clustering heat map **(B)** Gene ontology (GO) enrichment of upregulated genes in biological processes, cellular components, and molecular functions. **(C)** Kyoto Encyclopedia of Genes and Genomes (KEGG) enrichment analysis of upregulated genes. **(D)** Cross-linked genes involved in multiple functional collections related to phagocytosis and lipid recognition from the upregulated genes. **(E)** Left: Transcriptome verification experiment model diagram. Quantification number of iNKT cells engulfing lipid drops and the surface area of iNKT cells in contact with lipid droplets and the volume of engulfed lipid drops in aggregated iNKT clusters in untreated Cxcr6*^Gfp/+^* mice (n = 6) or mice treated with purified anti-mouse Fcgr4 antibodies. Right: Representative 3D images from ≥ 3 independent experiments showing aggregated iNKT cells (green) and TG drops (red, stained with Nile red) in the liver of Cxcr6*^Gfp/+^* mice fed MCD diet after purified Fcgr4 antibody treatment. 3D image dimensions (µm): X = 62 µm, Y = 62 µm, Z = 15 µm, where the XY plane is perpendicular to the objective lens. Representative 3D intersection images and single-cell cutting (after 3D surface rendering) showing the hepatic iNKT cell (green) ability for engulfing lipid drops (red). **(F)** After treatment with purified Fcgr4 antibody, mRNA levels of related genes involved in lipid treatment in iNKT cells decreased (n = 6). Data represent mean relative expression ± SEM. *P < 0.05; **P < 0.01; ***P < 0.001 using two-tailed unpaired student's t-test.

To characterize the functions of recruited iNKT cells, we isolated GFP+CD1d-α-GalCer-positive cells (iNKT cells) from MCS and MCD-fed Cxcr6*^Gfp/+^* mice by flow cytometry-assisted cell sorting and performed RNA sequencing analysis. We identified 1,206 genes differentially expressed in iNKT cells in the liver of mice fed with MCD and MCS diets. Among them, 184 genes were upregulated, and 1,022 genes were downregulated (Figure 6A). Further, we performed GO (gene ontology) enrichment of the differentially expressed genes in major functional classes of biological processes, cellular components, and molecular functions. The upregulated differentially expressed genes were significantly annotated in the processing of lipids and oxygenates, as well as in the biological processes regulating transport and bio-adhesion (Figure 6B, Left). Annotation of cellular components revealed that extracellular lysosomal and cytoplasmic vesicle functions were upregulated (Figure 6B, Middle). As for molecular functions, the upregulated differentially expressed genes were significantly annotated for serine hydrolase, serine-type peptidase, and peptidase activities; these enzymes promote decomposition of triglycerides into free fatty acids and glycerol (Figure 6B, Right). Also, the Kyoto Encyclopedia of Genes and Genomes (KEGG) enrichment analysis revealed that the upregulated differentially expressed genes were associated with phagosomes and FcγR-mediated phagocytic signaling pathways and enriched for signaling pathways of phospholipase D and lipid catabolism involving PI3K/AKT (Figure 6C).

Furthermore, among the upregulated differentially expressed genes, we screened for cross-linking genes involved in multiple functional categories related to phagocytosis and lipid recognition. From the interaction patterns of differentially expressed genes, we selected Fcgr4 (CD16.2) to verify its effect on lipid phagocytosis of iNKT cells (Figure 6D-E). We found that purified Fcgr4 antibody treatment severely affected lipid phagocytosis by iNKT cells in the cluster (Figure 6E), indicating that Fcgr4 played an important role in the iNKT cell phagocytosis. Additionally, after treatment with purified Fcgr4 antibody, mRNA levels of related genes (*Apoa4, Dgat2, Smpdl3b, Dpld1*) involved in lipid phagocytosis in iNKT cells decreased (Figure 6F). Collectively, these results indicated that the iNKT cell transcriptome recruited in the murine liver following consumption of MCD diet was significantly enriched in transcripts associated with lipid engulfing and processing.

### iNKT cell defect results in lipid metabolic disorder and exacerbates steatohepatitis

**Figure 7:**
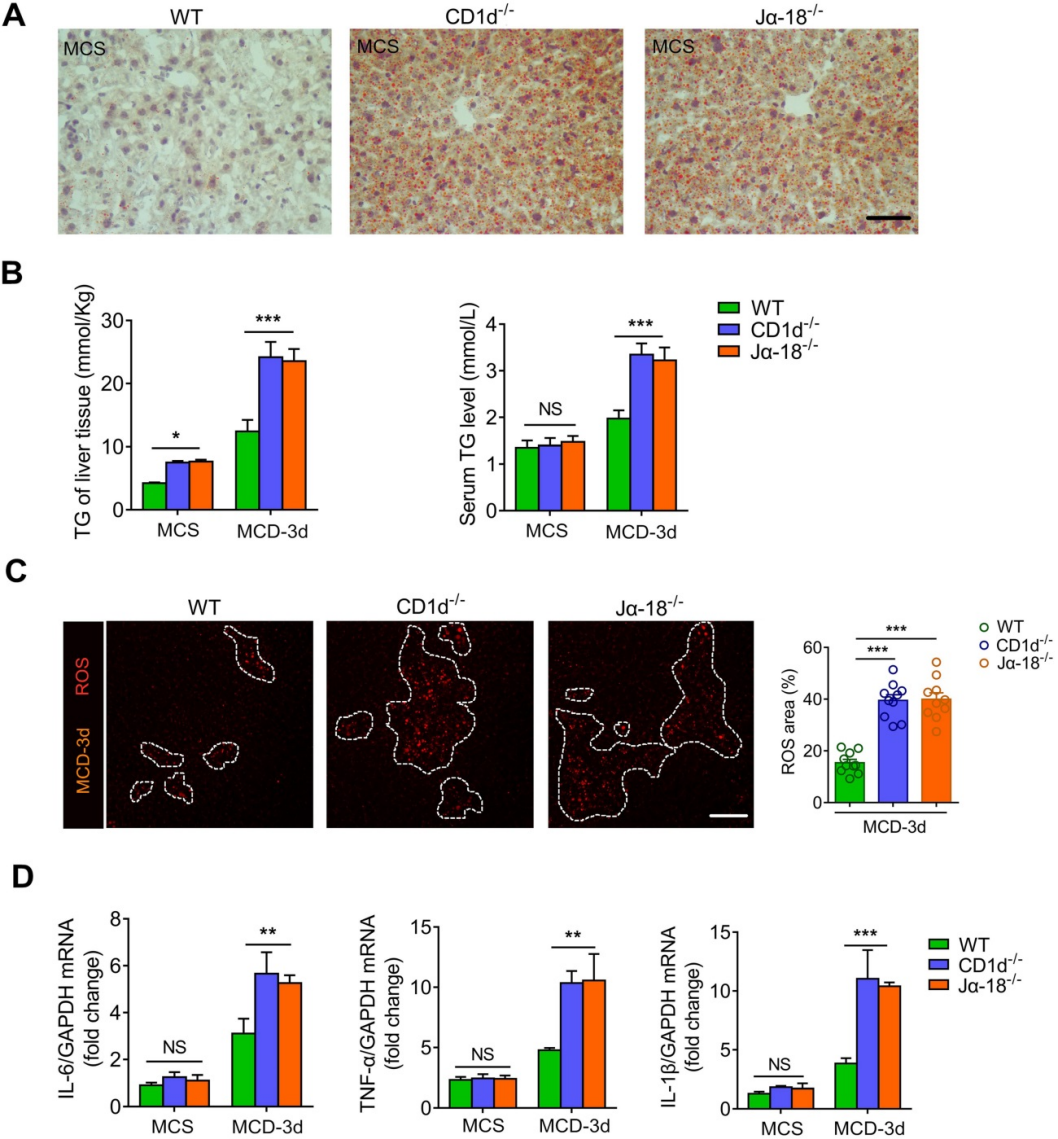
** iNKT cell defect results in lipid metabolic disorder and exacerbates steatohepatitis. (A)** Representative image of Oil Red O staining illustrating TG accumulation in liver tissues from WT, CD1d*^-/-^*, Jα18*^-/-^* mice fed MCS diet. Scale bar, 100 µm.** (B)** Quantitative analysis of intrahepatic TG concentrations and serum TG levels in WT, CD1d*^-/-^*, Jα18*^-/-^* mice (n = 5) fed MCS or MCD diet for 3 days. **(C)** Left: Representative images from ≥ 3 independent experiments intravital imaging snapshots of ROS (red) area (indicated with a white dashed line) in WT, CD1d*^-/-^*, Jα18*^-/-^* mice fed MCD diet for 3 days. Scale bar, 100 µm. Right: Quantitative analysis of ROS area in WT, CD1d^-/-^, and Jα18^-/-^ mice (n = 10) fed MCD diet for 3 days. **(D)** mRNA levels of inflammatory cytokines Il-6, Tnf-α, and Il-1β in liver tissues of WT, CD1d*^-/-^*, and Jα18*^-/-^* mice (n = 5) fed an MCS or MCD diet for 3 days. Data represent mean relative expression ± SEM. *P < 0.05; **P < 0.01; ***P < 0.001 using two-tailed unpaired student's t-test.

Several studies have reported that defects in iNKT cells cause lipid metabolism disorders in the liver 12, 42-44. We found that iNKT-deficient mice (CD1d^-/-^ and Jα18^-/-^ mice) had significantly higher liver triglyceride levels than WT mice at the same age (Figure 7A). When fed with MCD for 3 days, the triglycerides in the liver and peripheral serum of iNKT-deficient mice increased more than WT mice (Figure 7B). Besides, the oxidative stress component of mixed lipids released by hepatocyte necrosis increased significantly in iNKT-deficient mice (Figure 7C). Furthermore, we found that mRNA levels of inflammatory cytokines IL-6, TNF-α, and IL-1β in liver tissues of iNKT-deficient mice were significantly increased (Figure 7D). These results indicated that the defect of iNKT cells caused liver lipid metabolism disorder and aggravated steatohepatitis.

### Adoptive transfer of iNKT cells improves lipid accumulation level and inhibits fibrosis in Jα18^-/-^ mice

Currently, diet modification is a primary strategy for preventing steatohepatitis 45. In the present study, we altered the diet of mice with early steatohepatitis and found that the recruited aggregated iNKT cells gradually separated as the liver triglyceride levels decreased. Furthermore, when iNKT cell clusters disintegrated, the iNKT cells restored their original cell dynamics (Figure S8A-F).

**Figure 8:**
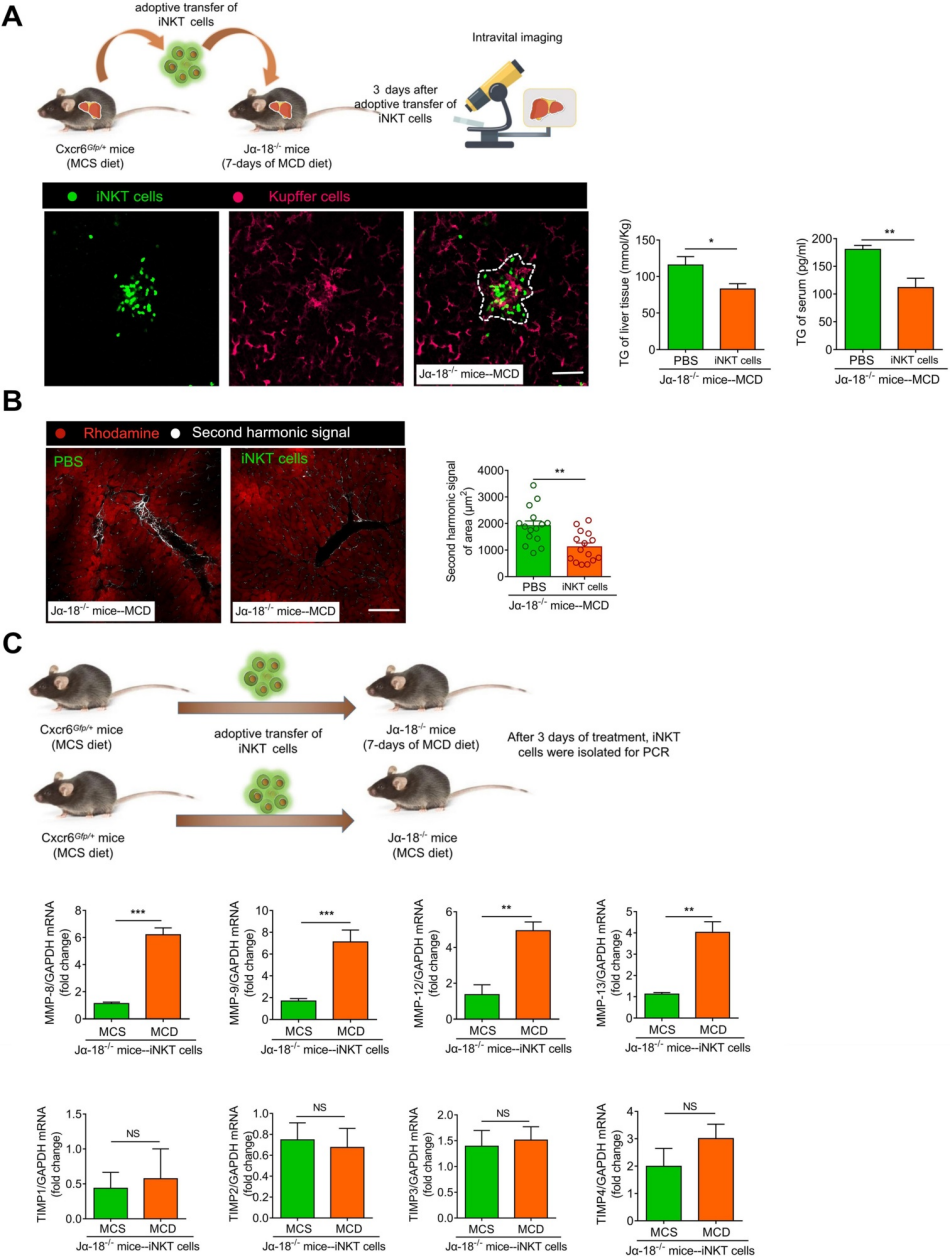
** Adoptive transfer of iNKT cells improves lipid accumulation and inhibits fibrosis in Jα18***^-/-^***mice. (A)** Left: Experimental model of adoptive transfer iNKT cells. Representative images were obtained from ≥ 3 experiments to examine the interactions between iNKT cells (green) and Kupffer cells (fuchsia, F4/80 antibody-labeled Kupffer cells) in Ja18*^-/-^*mice adoptively transferred with iNKT cells. Scale bar, 100 µm. Right: Quantitative analysis of intrahepatic TG concentrations and serum TG levels in Ja18*^-/-^*mice (n = 5) adoptively transferred with iNKT cells or PBS.** (B)** Left: Representative images were obtained from ≥ 3 experiments to examine the second harmonic signal (white) in Ja18*^-/-^*mice adoptively transferred with iNKT cells or PBS, liver leaflet outline visualized by Rhodamine (red) markers. Scale bar, 100 µm. Right: Quantitative analysis of second harmonic signal of area in Ja18*^-/-^*mice (n = 5) adoptively transferred with iNKT cells or PBS. **(C)** Experimental model of adoptive transfer iNKT cells. Quantitative mRNA level analysis of MMP-8, MMP-9, MMP-12, MMP-13, TIMP1, TIMP2, TIMP3, and TIMP4 from iNKT cells adoptively transferred into MCS or MCD fed Ja18*^-/-^*mice (n = 5). Data represent mean relative expression ±SEM. *P < 0.05; **P < 0.01; ***P < 0.001 using two-tailed unpaired student's t-test.

However, it is unclear if the iNKT cells have a therapeutic effect in severe steatohepatitis or steatohepatitis together with fibrosis. To verify iNKT cell clusters' protective role in long-term MCD-induced (7 days) steatohepatitis, we adoptively transferred 1×10^6^ iNKT cells to Jα18^-/-^ mice (MCD-7 days). The control group comprised of adoptively transferred PBS treatment for 3 days. On the third day of the adoptive transfer of iNKT cells, we used live microscopy and observed that iNKT and Kupffer cell aggregation characteristics reappeared in Jα18^-/-^ mice (Figure 8A, Video S10). We also found that the triglyceride levels in the liver and peripheral circulation of Jα18^-/-^ mice in the iNKT cell treatment group were significantly lower than in the control group (Figure 8A). Furthermore, compared to the controls, the occurrence and development of fibrosis in the hepatic parenchyma of Jα18^-/-^ mice in the treatment group were suppressed (Figure 8B, Video S11). Additionally, the collagen deposition and CK19 expression in the hepatic parenchyma of Jα18^-/-^ mice in the treatment group were considerably decreased compared to the controls (Figure S9A-B). Moreover, hepatic hydroxyproline, Col1a1, and Col1a2 gene expressions in the liver also showed a significant reduction following treatment with iNKT cells (Figure S9C-D). To confirm that adoptively transferred iNKT cells could inhibit liver fibrosis, iNKT cells after adoptive transfer of Jα18^-/-^ mice (MCS or MCD) were collected. The expression levels of matrix metalloproteinases (MMP), MMP-8, MMP-9, MMP-12, and MMP-13, involved in eliminating the extracellular matrix, were increased, while the expression levels of tissue inhibitor of metalloproteinases (TIMP) TIMP1, TIMP2, TIMP3, and TIMP4, involved in inhibiting the elimination of extracellular matrix, did not change significantly (Figure 8C). The adoptively transferred iNKT cells also caused markedly high expression of IL-10 and IFN-γ (Figure S9E). These results indicated that the adoptive transfer of iNKT cells had a significant therapeutic effect on lipid accumulation and played an important role in inhibiting fibrosis.

## Discussion

As a research technology, Intravital imaging has been widely used in the research of a variety of diseases, such as liver transplantation, liver injury and the treatment of vascular inflammation 46-49. In addition, intravital imaging is also a powerful tool to study the dynamic interactions between immune cells. During the initiation of an immune response to steatohepatitis, a variety of immune cells interact with each other, most of them through mutual contacts between cells. The biological effects of this cross-talk regulate the immune response of the liver. Previously, Kubes et al. used live imaging and described the recruitment of multiple immune cells in the early inflammatory response to aseptic liver injury 22-24; Gustavoet al. also used live imaging to locate neutrophils in drug-induced liver injury 50. The authors found that the cells migrated to DNA-rich regions, showing that DNA accumulation in the liver is a characteristic and novel feature in DILI pathogenesis.

In this study, we visualized, for the first time, the initiation and development of inflammation during non-alcoholic fatty liver disease. Our results showed that the inflammation in non-alcoholic fatty liver begins as local inflammation. The iNKT and Kupffer cells clustered together and interacted in an independent pathological microenvironment to remove free lipids released by necrotic liver cells. The adoptive transfer of iNKT cells to Jα18^-/-^ mice further confirmed the role of iNKT and Kupffer cell clusters to balance the liver and peripheral lipid levels and inhibit fibrosis during steatohepatitis. We employed the multi-photon *in vivo* imaging, revealing the dynamic interaction between iNKT and Kupffer cells in cell clusters during early steatohepatitis.

The iNKT lineage is a highly specialized subset of innate lymphoid cells that recognize lipid antigens, are abundant in the liver, and represent 30-50% of lymphocytes 13. Previous studies have shown that the iNKT cells are recruited to the liver in large numbers during HFD or MCD-induced NASH. Upon activation, iNKT cells produce increased IFN-γ and IL-4, facilitating interaction with helper T cells 1 (Th1) or 2 (Th2) in the liver microenvironment 33, 51. Although the recruitment of iNKT cells to the liver is well recognized, limited information is available on the distribution of the recruited iNKT cells, their specific behavioral changes, and potential roles in the development of steatohepatitis. Similar to the previous reports of chemotaxis of neutrophil recruitment and the appearance of "clustering" 52, 53, our results showed that the distribution of recruited iNKT cells in the liver was not uniform. After extravasation from blood vessels, iNKT cells showed extremely coordinated chemotaxis and “clustering” phenomenon, similar to the dense “swarming” behavior of insects. During steatohepatitis development, multiple small clusters were bridged into large clusters, as shown by intravital imaging. Furthermore, the dynamics of the clustered iNKT cells was altered. Long-range migration of cells and short-range communication by local chemical signaling and by cell-cell contacts are vital to the control of an immune response, as previously described 54, 55. In our study, the liver iNKT cells randomly move in the hepatic sinusoids to play an immune surveillance function in basic physiological conditions. However, the limited in iNKT cell motility are related to the complex components released by necrotic hepatocytes in the pathological microenvironment, which may contain a variety of chemokines or lipid components that can activate aggregated iNKT cells in the steatohepatitis. The normally-moving iNKT cells in the liver quickly stop moving when activated by α-GalCer, as previously described 34. Obviously, the clustered iNKT cells are activated and transformed from immune surveillance function to lipid clearance function in steatohepatitis. During UA intervention, the oxidative stress in hepatocytes was alleviated, thereby preventing necrosis caused by excessive lipid accumulation. Hence, the recruited iNKT cells in the liver showed uniform distribution rather than clustering. We also found that UA treatment improved endothelial dysfunction by inhibiting necrosis caused by oxidative stress in the liver cells.

As revealed by intravital imaging studies, the most significant finding of our study is that the iNKT cells engulfed the free lipids released by necrotic hepatocytes. This lipid phagocytic function of iNKT cells in steatohepatitis has not been reported previously. Although the Kupffer cells in the clusters also play a role in removing lipids, we focused on the lipid clearing ability of iNKT cells. Phagocytosis involves the engulfing of particles by the cell membrane to form phagosomes, which fuse with primary lysosomes to form the phagolysosomes containing multiple hydrolases 56. Our transcriptome studies revealed the functional changes of the recruited iNKT cells spanning from immune monitoring to phagocytosis and lipid metabolism. In the steatohepatitis microenvironment, the upregulated, differentially expressed genes of iNKT cells were significantly enriched in enzymes of the lysosomes and phagosomes, as well as various hydrolases and peptidases necessary for lipid metabolism.

The upregulated, differentially expressed genes were also enriched in C-type lectin receptors and Fc receptors, both of which are phagocytic receptors 57, including the potent Fcgr4 gene involved in the Fc phagocytic receptor function. It has been reported that the binding of Fcgr4 to IgE could promote macrophage-mediated phagocytosis 58. Similarly, iNKT cell transcriptome sequencing showed that the upregulated Fcgr4 bound to IgE. Furthermore, Fcgr4 was significantly annotated in cell membrane-like structural functions and collections that exhibit a positive recognition response to lipids or oxygenates. We also found that the CD300 gene, which regulates immune cell processes, was significantly up-regulated (data not shown). Previous studies have shown that CD300 molecules are involved in the formation of Fc receptors and can recognize lipids exposed on the outer leaflets of dead cell plasma membranes, such as extracellular ceramides, phosphatidylserine, and phosphatidylethanolamine 59.

Moreover, the genes involved in cellular lipid catabolism were significantly upregulated which included apolipoprotein A-IV (*Apoa4*) 60, sphingomyelin phosphodiesterase acid-like 3 (*Smpdl3b*) 61, 62 and lysophospholipase D (*Gdpd1*) 63. Among the downregulated, differentially expressed genes, those involved in phagocytosis were not significantly enriched. Therefore, in early NASH, iNKT cells underwent a profound transcriptional change upon encountering lipid antigens and were temporarily activated. Also, we found that the lipid load inside the iNKT cells increased, largely affecting other biological functions of iNKT cells. This could be an important factor contributing to fewer upregulated differentially expressed genes compared to the number of downregulated genes and indicated lipid processing to be the main function of recruited iNKT cells.

Most immune cells, including T cells, macrophages, and NK cells, display some pliability upon encountering different environments or antigens 64. These immune cells are distinguished by the signature transcription factors they express and the predominant production of cytokines they produce. Similarly, iNKT cells of the thymus can be categorized into populations defined by transcriptional factors and cytokines, including iNKT1 (T-bet/IFN-γ), iNKT2 (Gata-3/IL-4), and iNKT17 (Ror-t/IL-17) 65, 66. However, there is evidence that there are other functional subsets of iNKT cells in peripheral tissues 67-69. In our study, unlike the classical α-GalCer-activated iNKT cells, the iNKT cells in the cluster showed an activation pattern with high IFN-γ expression levels. Previous studies have found that activated iNKT cells accumulated and released IFN-γ in the liver to prevent neutrophil infiltration and protected the organ from injury by accelerating liver neutrophil apoptosis 70. In diet-induced obesity and metabolic disorders, IFN-γ reduced insulin signaling and lipid storage in fat cells 71. Similarly, recent studies have shown that the iNKT cells recognized and responded to bacterial antigens and participated in bacterial clearance; the adoptive transfer of iNKT cells greatly reduced the bacterial load in mice, and the IFN-γ produced by iNKT cells was crucial in this process 72-74.

Based on previous studies, we speculated that the high expression level of IFN-γ in the iNKT cell cluster might promote the removal of free lipids. Previous studies alsodemonstrated that IL-10-producing iNKT cells with a regulatory potential (NKT10 cells) represented a distinct iNKT cell subset in peripheral tissues; iNKT cells pretreated with α-GalCer could produce and secrete the immunomodulatory cytokine IL-10 12. In our study, iNKT cells recruited without α-GalCer pretreatment also exhibited high IL-10 expression. It is possible that in the complex pathological microenvironment of steatohepatitis, iNKT1 (T-bet/IFN-γ) subsets are recruited into clusters. However, we could not exclude the possibility of potential participation of iNKT-IL-10 cells or other iNKT subsets.

During the immune response-cell interaction in NASH, the pathological microenvironment encountered by the immune cell cluster also plays a critical role in macrophage polarization and inflammatory response. Kupffer cells can be divided into two types of polarization according to their phenotypes and secreted factors, namely classical polarization M1 type (pro-inflammatory) and selective polarization M2 type (anti-inflammatory) 41. A significant aspect of our study is that it revealed the interactive dialogue between iNKT and Kupffer cells and visualization of detailed biological effects. The Kupffer cells in the normal physiological state adhered to the Disse cavity of hepatic sinusoids with silk-like or plate-like pseudopods. Conversely, during steatohepatitis, the Kupffer cells formed clusters and were deprived of normal villi-like or finger-like protrusions, as observed in the high-resolution 3D imaging.

The high expression level of the non-polymorphic MHC class I molecule CD1d by Kupffer cells in the cluster is an important characteristic feature that can provide iNKT cells with more opportunities to present glycolipid antigens 75. Previous studies have shown that iNKT cells recognize glycolipid antigens presented by CD1d. One of the most thoroughly studied glycolipids capable of activating iNKT cells is α-GalCer, a compound derived from marine sponges 76. However, since α-GalCer cannot be synthesized in humans or mice, it is not a physiological ligand for iNKT cells. Most of the naturally occurring exogenous iNKT cell antigens are derived from microorganisms. Recent work has shown that iNKT cells directly recognize α-linked sphingolipids and diacylglycerol antigens expressed by bacteria like Sphingomonas, Ehrlichia, and Borrelia burgdorferi in a CD1d-dependent manner 77, 78. However, in this study, we could not determine if the contents released by necrotic hepatocytes contained α-linked sphingolipids and diacylglycerol antigens or other endogenous glycolipid antigens that could potentially activate iNKT cells.

During the onset of steatohepatitis, stress cells and necrotic cells release a variety of lipid and non-lipid components, including triglycerides and chemokines, triggering damage-associated molecular patterns (DAMPs). A series of complex interactions between multiple components causes iNKT cells to be chemotactically clustered and activated. Previous studies found that peroxisome-derived lipids (ether-bonded lipids) are self-antigens that stimulate iNKT cells in the thymus and peripheral organs 79. Therefore, the possibility of direct or indirect activation of iNKT cell by triglyceride conjugates or oxidized triglyceride components cannot be ruled out during the development of steatohepatitis. Also, the free lipid component in the liver contains many other entities in addition to triglycerides and, despite their low content, most likely activate iNKT cells. The levels of various lipid metabolic intermediates in the local microenvironment play a crucial role in determining the fate of immune cells. We also found that early steatohepatitis could be reversed by altering the diet. However, due to the lack of specific markers for clustered iNKT cells, the fate of iNKT cells following the disintegration of cell clusters could not be clarified.

Another significant finding of our study is that the adoptive transfer of iNKT cells promoted the reduction of liver and peripheral triglyceride levels and alleviated fibrosis. Using a multi-photon microscope for second harmonic signal imaging, we successfully visualized the degree of liver fibrosis. We also found that Jα-18^-/-^ mice were more likely to develop MCD-induced steatohepatitis and early fibrosis. Evidently, the iNKT cells may directly or indirectly inhibit hepatic stellate cell activation or accelerate extracellular matrix clearance through autocrine and paracrine cytokines. As previously reported, NK and NKT cells secrete IFN-γ to induce the apoptosis of activated HSCs, thereby exerting anti-fibrotic effects 80-83. MMP is the primary enzyme system that degrades the extracellular matrix 84, and TIMP prevents the degradation of the extracellular matrix by inhibiting MMP, thereby promoting liver fibrosis 84, 85. Although we lacked the visual evidence to prove that iNKT cells directly cleared the extracellular matrix, we found that the MMP/TIMP ratio of the iNKT cells increased following adoptive transfer. Thus, iNKT cells were directly involved in the extracellular matrix clearance in steatohepatitis. Since the current understanding of liver immunotherapy is mainly based on the research strategy of static nodes, it is difficult to uncover the dynamic details of the immunotherapy process. However, *in vivo* visual observation of iNKT cells' behavior in clusters to clear free lipids and extracellular matrix after infusion in steatohepatitis mice has provided a better understanding of the unique immune response of the liver. Exploring the key events in steatohepatitis treatment and the temporal and spatial dynamic information on multi-cell participation is of considerable significance and identifies target cells or proteins for blocking the occurrence and development of steatohepatitis.

In conclusion, our study has demonstrated that iNKT cells in the liver play a crucial role in clearing free lipids in the inflammatory microenvironment observed during early steatohepatitis. The pathogenesis of steatohepatitis, a complex metabolism-associated disease, involves many different cell types and represents an enormous challenge to develop novel therapeutic targets. However, our current findings suggest the clinical application of iNKT cells as a promising steatohepatitis treatment strategy. In the future, we plan to perform in-depth visualization experiments to study the phagocytosis mechanism in iNKT cells and to find endogenous lipid antigens that can drive iNKT cells to induce self-reactivity. Identifying the class of iNKT subtypes highly sensitive to steatohepatitis lipids that regulate iNKT cell functions would be significant in treating other pathological conditions caused by lipid accumulation, such as inflammation of the adipose tissue. This will provide new research ideas and methods for the clinical prevention and treatment of diseases associated with lipid accumulation.

## Supplementary Material

Supplementary figures and tables.

Supplementary video/movie S1-S11.
