# Supplementary material for: Intravital imaging of interactions between iNKT and kupffer cells to clear free lipids during steatohepatitis
Source: Theranostics. 2021 Jan 1;11(5):2149–69. doi: 10.7150/thno.51369 (PMC7797696; doi:10.7150/thno.51369)
Supplement: Supplementary file 1 — Supplementary figures and tables. [file thnov11p2149s1.pdf]

Supplementary Material

Liver histopathology and immunohistochemistry

Deeply anesthetized mice were intravenously perfused with ice-cold phosphate-buffered saline (PBS), after which liver tissues were collected, embedded in optimal cutting temperature compound, and sliced into 10 μm thick sections. The sections were stained using the Lipid Oil Red O Staining Kit (CAS# MAK194; Sigma-Aldrich, St. Louis, MO). For histopathological examination, the livers were fixed in 10% formalin, embedded in paraffin, sectioned, and stained with hematoxylin and eosin (H&E, ab245880, Abcam, UK) and Sirius Red (CAS# 2610-10-8; Sigma-Aldrich, St. Louis, MO) stain. To estimate the extent of necrosis or collagen deposition, the specimen was observed under a light microscope. Image J was used to calculate the necrosis area. The liver sections were CK19 (1:500, ab15463, Abcam, UK) stained and the specimens were observed under a light microscope. Images were obtained at×20 magnification fields from each sample.

Biochemical analyses

Alanine or aspartate transaminase (ALT or AST) activities, hepatic hydroxyproline, and hepatic triglyceride (TG) levels were quantified using commercial kits (Jiancheng Tech, Nanjing, China), according to the manufacturer's instructions.

RNA sequencing and analysis

Natural Killer (NK) cells were depleted by injecting 10 μg of purified anti-GM1 24 h before feeding MCS or MCD. Cxcr6$^{Gfp/+}$ transgenic mice were fed MCS or MCD diets. On day 3, the green fluorescent protein (GFP)$^{+}$ CD1d-α-Galcer^{+} iNKT cells from the liver were sorted directly into TRIzol (Invitrogen, Carlsbad, CA). mRNA libraries were prepared from polyA^{+} RNA using the Whole Transcriptome Amplification Sequencing Technology SEQR Kit (Sigma-Aldrich). Gene analysis was performed using the DESeq2 package of R software (Version 1.20.0), and the data were normalized using the default median-ratio method. Differential expression of genes was considered significant at a linear fold-change threshold of 2 and a Q value of 0.001.

Isolation and culture of primary liver cells

Mouse hepatocytes were isolated using a 2-step collagenase perfusion method. The cells were gently dispersed into DMEM High Glucose Medium (DMEM, hyclone, USA).and filtered through a 100 μm pore size cell strainer to remove connective tissues and undigested tissue fragments. The cells were centrifuged at 50 g for 3 min at 4℃ and re-suspended in 20 mL DMEM High Glucose Medium. Subsequently, 20 mL of 90% Percoll (GE Healthcare Life Sciences, Sweden) in PBS was added to the cells and centrifuged at 200 g for 10 min at 4℃. The viable cell pellet from the bottom of the Percoll gradient was taken up in 30 mL of warm DMEM High Glucose Medium. Cells were cultured in DMEM, containing 10% fetal bovine serum (FBS, Gibco, Gaithersburg, MD, USA) and 1% penicillin/streptomycin and incubated at 37℃.

Cell viability measurement using CCK-8

Hepatocytes were cultured in a 96-well plate with iNKT cells for 12 h. Subsequently,10 μL of CCK-8 (Dojindo Laboratories, Japan) solution was added to each well, and the plate was incubated at 37℃ in a 5% CO_2 incubator for up to 2 h. The 96-well plate was placed in a microplate reader, and the optical density of each well was measured at 450 nm.

Real-time RT-PCR

Total RNA was extracted from iNKT or Kupffer cells from liver tissues using TRIzol reagent. Aliquots (2 μg) of total RNA were reverse transcribed using SuperScript II reverse transcriptase (Thermo Fisher Scientific, Waltham, MA). Real-time polymerase chain reaction (PCR) was performed using the IQ SYBR Green SuperMix Reagent (Bio-Rad Laboratories, Hercules, CA, USA) on a real-time PCR machine (CFX96, Bio-Rad, USA) according to the manufacturer's instructions. For quantification, the comparative cycle threshold (Ct) method was used. The Ct values for each gene were normalized to the Ct value of glyceraldehyde-3-phosphate dehydrogenase (GAPDH) in the same cDNA samples based on the $2^{-\Delta\Delta Ct}$ method. Expression levels of the target genes were normalized to those of GAPDH in each sample. Primers sequences are presented in Table S1

Supplementary Figures and Figure Legends

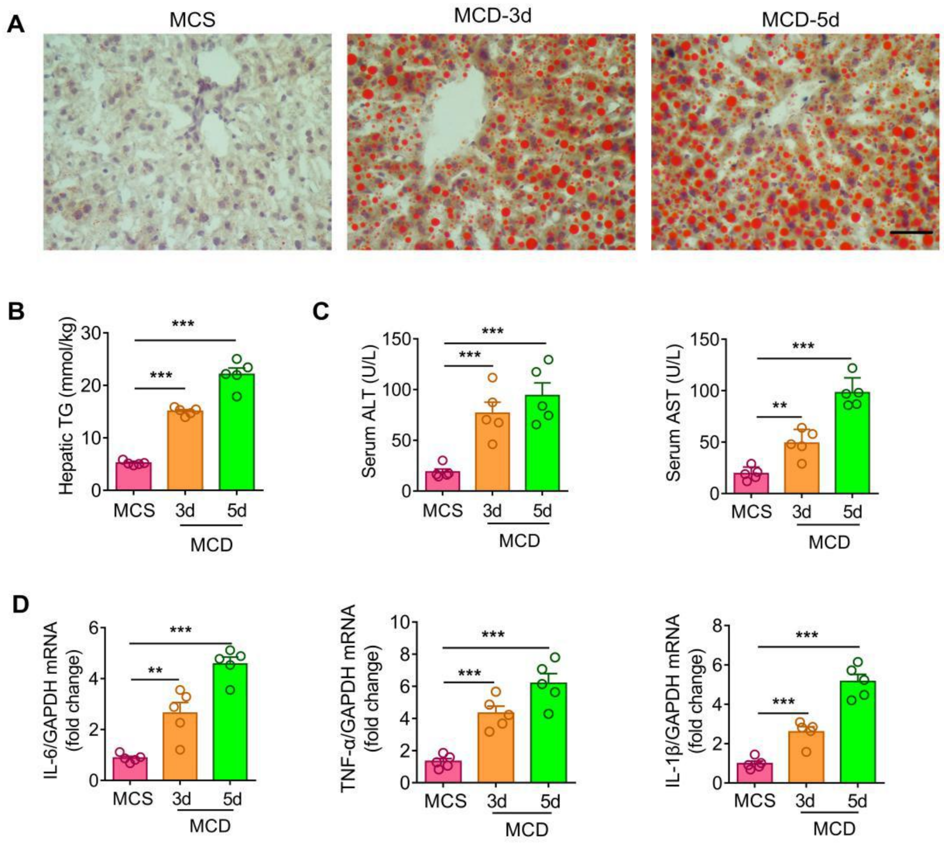

Figure S1. The liver of WT mice showed NASH characteristics under short-term induction with MCD. WT mice (n = 5) were fed MCS or MCD diet for 3 or 5 days. **(A)** Representative image of Oil Red O staining illustrating TG accumulation in liver tissues. Scale bar, 100 μm. **(B, C)** Quantitative analysis of intrahepatic TG concentrations **(B)** and serum ALT and AST levels **(C)** **(D)** mRNA levels of inflammatory cytokines Il-6, Tnf-α, and Il-1β in liver tissues. Data represent mean relative expression ± SEM. *P< 0.05; **P< 0.01; ***P< 0.001 using two-tailed unpaired student's t-test.

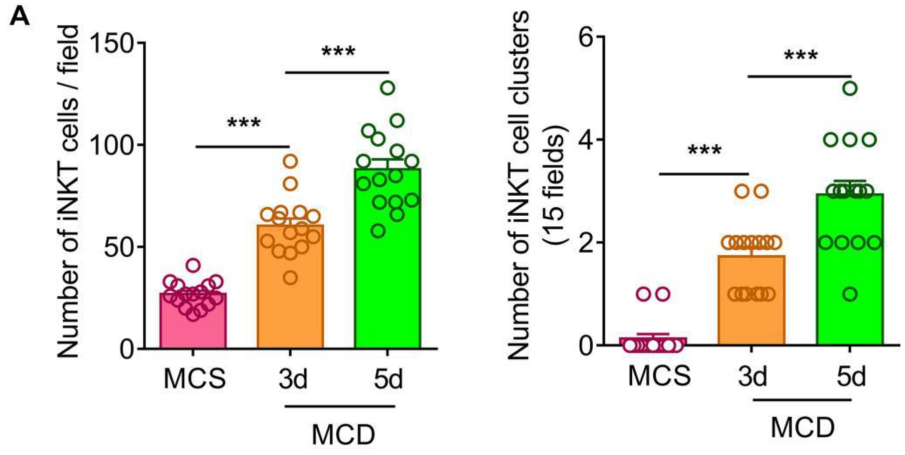

Figure S2. Quantification of the number of recruited iNKT cells and iNKT cell clusters in Cxcr6$^{Gfp/+}$mice (n = 3) fed MCS or MCD diet for 3 or 5 days. Data represent mean relative expression ± SEM. *P< 0.05; **P< 0.01; ***P< 0.001 using two-tailed unpaired student's t-test.

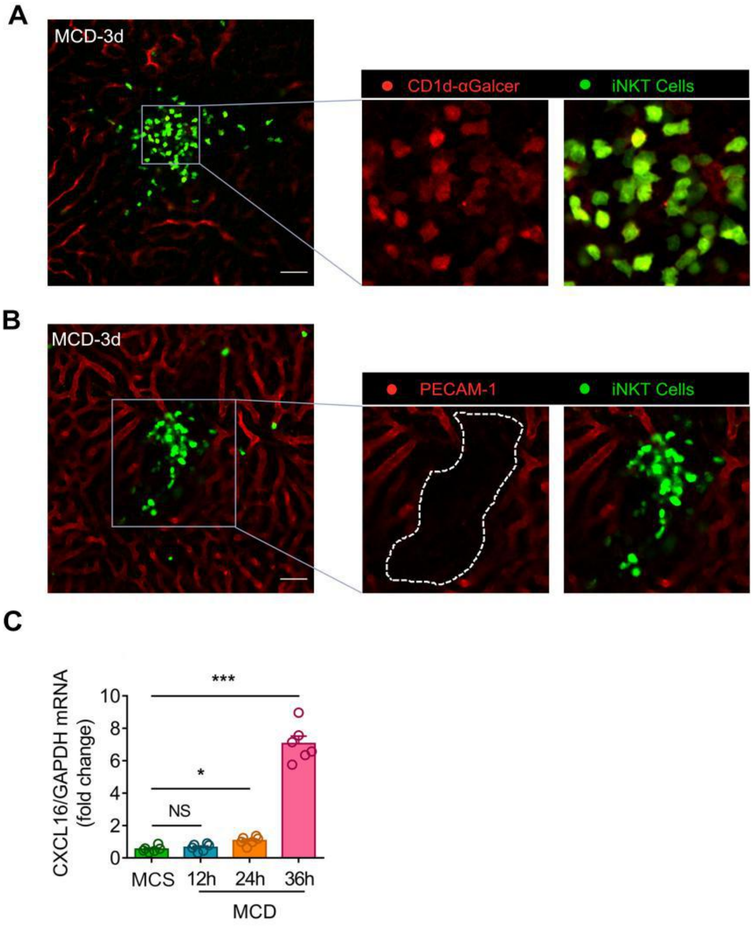

Figure S3. Cxcr6$^{Gfp/+}$ mice fed MCD diet for 3 days were subjected to hepatic intravital microscopy, and the change in CXCL16 mRNA level was detected. (A) Representative images from 3 independent experiments of intravital imaging snapshot iNKT cells (green) co-localized with CD1d-α-Galcer (red). Scale bar, 100 μm. **(B)** Representative intravital imaging snapshot showing fluorescence continuity of endothelial cells (Alexa Fluor 647-conjugated anti-PECAM-1, red) in the iNKT cell cluster (green) was interrupted or the fluorescence blur disappeared. Scale bar, 100 μm. (C) mRNA levels of chemokine CXCL16 in mice fed MCS or MCD diet. Data represent mean relative expression ± SEM. *P< 0.05; **P< 0.01; ***P< 0.001 using two-tailed unpaired student's t-test.

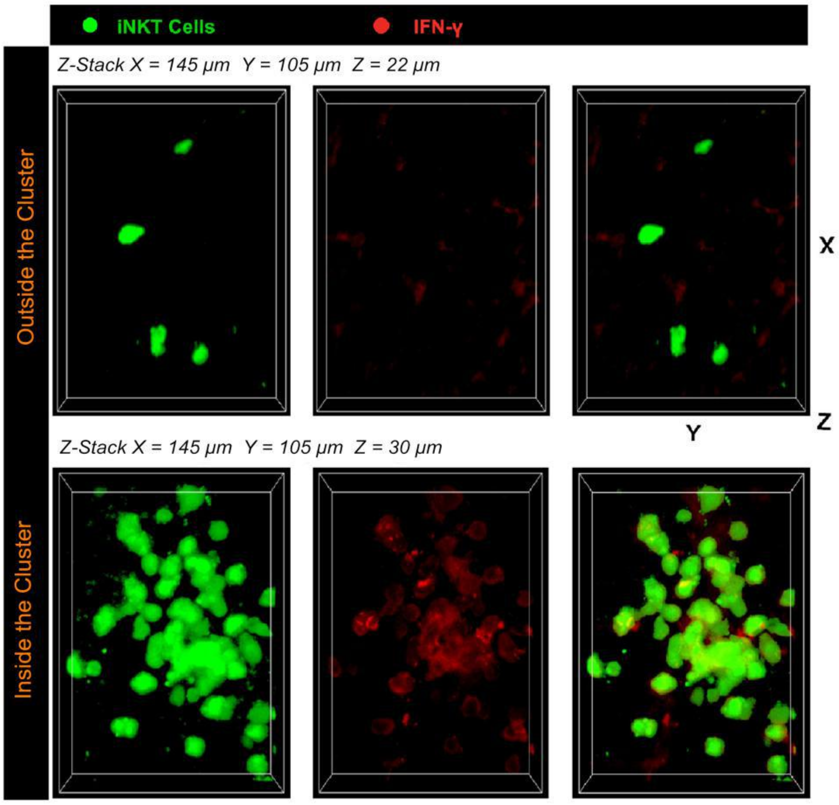

Figure S4. High expression of IFN-γ in aggregated hepatic iNKT cells after feeding MCD diet. Representative images from 3 independent experiments showing internal or external 3D colocalization of clusters (blue 3D Render) of iNKT cells with IFN-γ (red, labeled with Alexa Fluor 647-conjugated anti-IFN-γ antibodies) in Cxcr6$^{Gfp/+}$ mice fed MCD diet for 3 days. Image dimensions, X = 145 µm, Y = 105 µm, Z = 22 µm or 30 µm, where the XY plane is perpendicular to the objective lens.

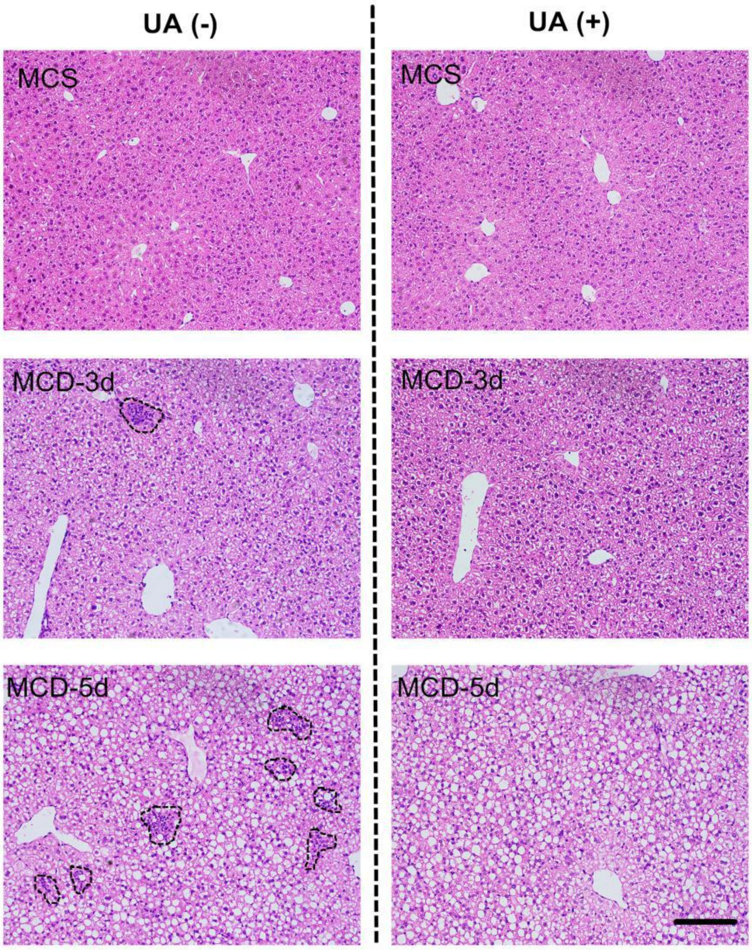

Figure S5. Uric acid prevented hepatic necrotizing inflammatory lesions in MCD-induced early steatohepatitis. Representative image of H&E staining and the dotted line is the area of hepatocyte necrosis and inflammation. Scale bar, 100 μm.

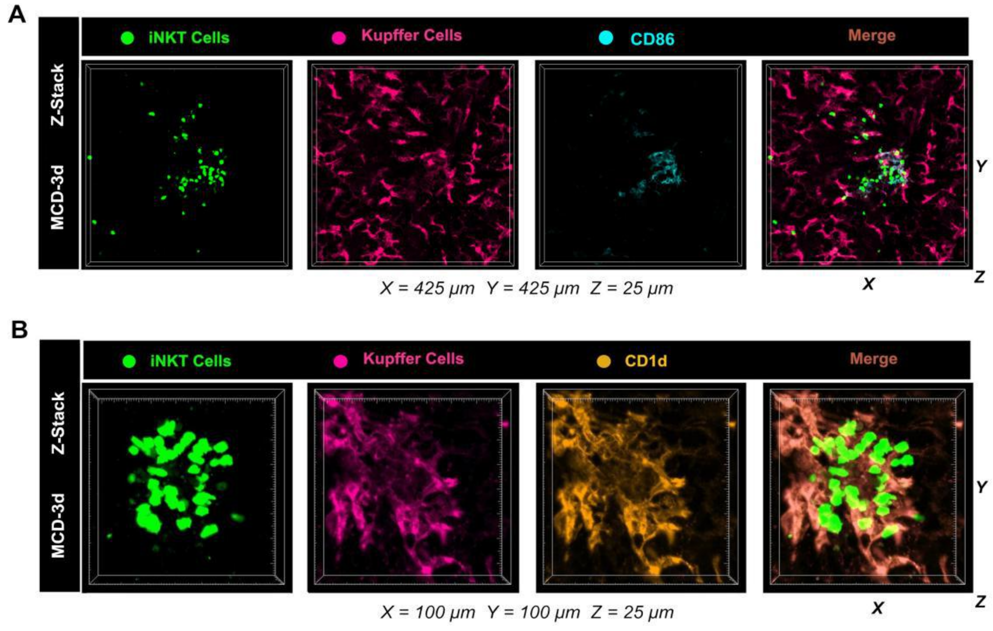

Figure S6. Kupffer cells in clusters were subjected to hepatic intravital microscopy in Cxcr6$^{Gfp/+}$ mice fed MCD diet. (A) Representative images were obtained from ≥ 3 experiments to examine the expression of CD86 (yellow, PE-anti-mouse CD86 antibody) in clusters of iNKT cells (green) and Kupffer cells (Fuchsia, Alexa Fluor 647-conjugated anti-mouse F4/80 antibody). 3D images of iNKT cells and Kupffer cells obtained by Z-stacking were scanned. X = 425 μm, Y = 425 μm, Z = 25 μm, where the XY plane is perpendicular to the objective lens. **(B)** Representative images were obtained from ≥ 3 experiments to examine the expression of CD1d (yellow, PE-anti-mouse CD1d antibody) in clusters of iNKT cells (green) and Kupffer cells (Fuchsia, Alexa Fluor 647-conjugated anti-mouse F4/80 antibody). 3D images of iNKT cells and Kupffer cells obtained by Z-stacking were scanned. X = 100 μm, Y = 100 μm, Z = 25 μm, where the XY plane is perpendicular to the objective lens.

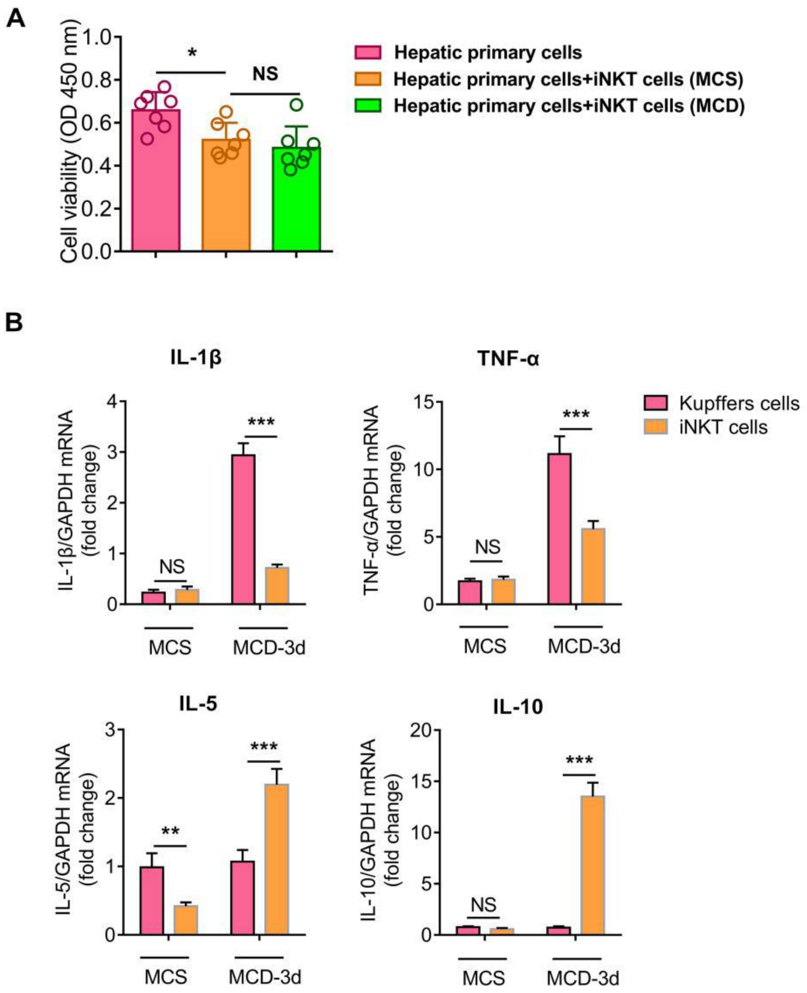

Figure S7. Effect of iNKT cells in different states on the viability of liver cells and the ability of iNKT and Kupffer cells to promote or inhibit inflammation. (A) Cell viability of primary liver cells was detected by the CCK8 assay after treatment with iNKT cells in different states for 12 h. **(B)** Quantitative analysis of mRNA levels of TH1/2 cytokine (TH1: IL-1β and TNF-α; TH2: IL-5 and IL-10) mRNA levels in iNKT and Kuffer cells under MCS or MCD-induced conditions. Data represent mean relative expression ± SEM. *P< 0.05; **P< 0.01; ***P< 0.001 using two-tailed unpaired student's t-test.

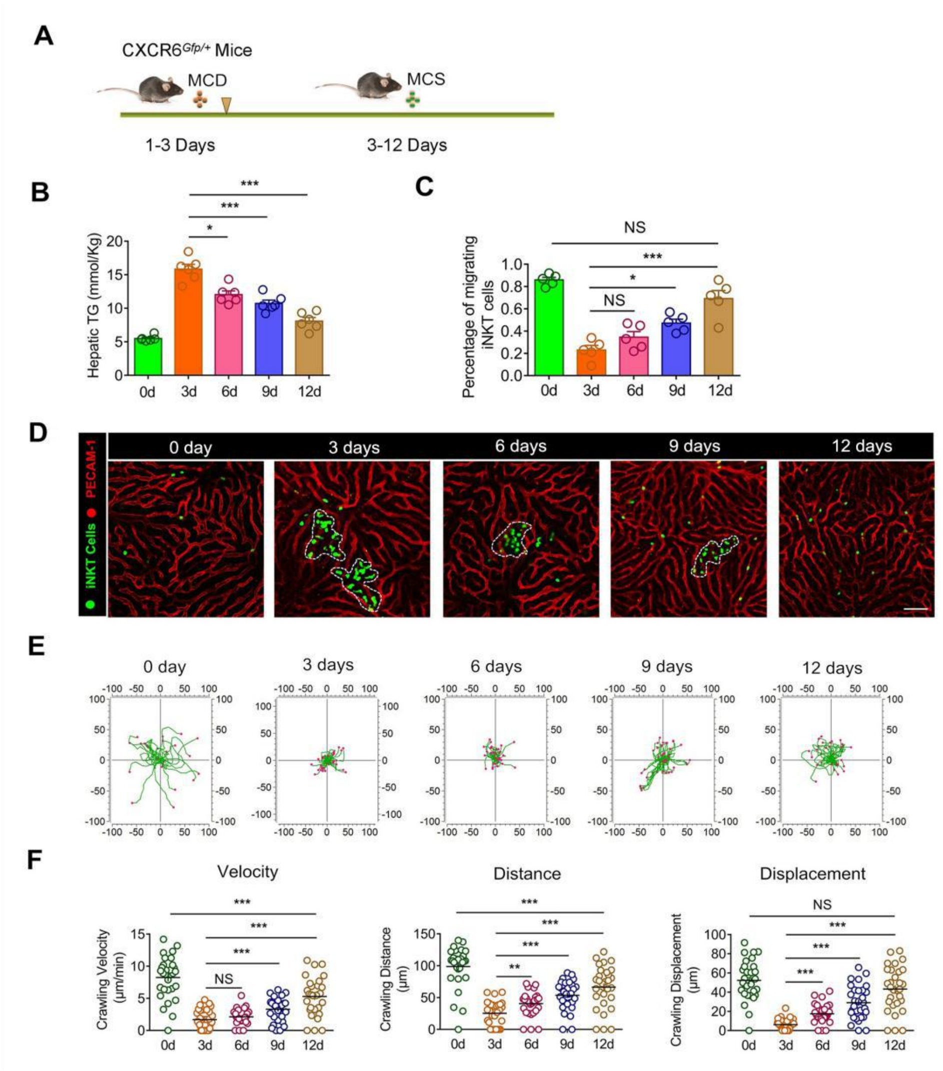

Figure S8. Migratory ability of aggregated iNKT cells was restored after the dissipation of hepatic triglyceride (TG) accumulation. (A) Cxcr6$^{Gfp/+}$ mice (n = 6) were fed MCD diet for 3 days, then fed MCS diet for an additional 9 days. **(B)** TG concentrations were quantified at different time points to evaluate the lipid accumulation levels (n = 6). **(C)** Quantification of migrating iNKT cells in aggregated clusters of Cxcr6$^{Gfp/+}$ mice (n = 5) at different time points. **(D)** Representative intravital imaging snapshots show changes in iNKT cell (green) aggregation in the livers of Cxcr6$^{Gfp/+}$ mice (n = 5) at different time points. Scale bar, 100 μm. The sinusoidal endothelium was labeled using Alexa Fluor 647 anti-platelet endothelial cell adhesion molecule (PECAM-1) antibodies (red). iNKT cell clusters are indicated with a white dashed line. **(E)** Overlay of iNKT cell migratory tracks was plotted after aligning their starting position. Data were collected from three mice, and each scan was imaged for 10 min. **(F)** Scatter plots of the velocity,

distance, and displacement of migrating iNKT cells within Cxcr6$^{Gfp/+}$ mice (n = 6) at different time points. Thirty migrating iNKT cells from three independent experiments were pooled. Data represent mean relative expression ± SEM. *P< 0.05; **P< 0.01; ***P< 0.001 using two-tailed unpaired student's t-test.

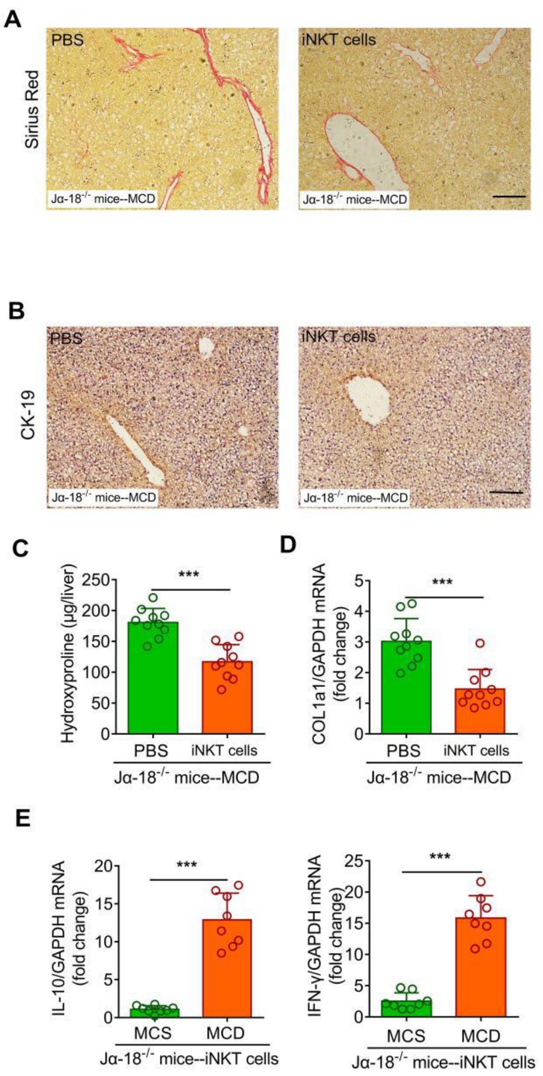

Figure S9. Adoptive transfer of iNKT cells improved the level of fibrosis in Jα18$^{-/-}$ mice. (A) Left: Representative images were obtained from ≥ 3 experiments to examine the collagen deposition (Sirius Red) in Ja18$^{-/-}$ mice adoptively transferred with iNKT cells or PBS. Scale bar, 100 μm. Right: Quantitative analysis of collagen deposition in Ja18$^{-/-}$ mice (n = 5) adoptively transferred with iNKT cells or PBS. **(B)** Left: Representative images were obtained from ≥ 3 experiments to examine the CK19 expression in Ja18$^{-/-}$ mice adoptively transferred with iNKT cells or PBS. Scale bar, 100 μm. Right: Quantitative analysis of CK19 expression in Ja18$^{-/-}$ mice (n = 5) adoptively transferred with iNKT cells or PBS. **(C)** Quantitative analysis of hydroxyproline in Ja18$^{-/-}$ mice (n = 5) adoptively transferred with iNKT cells or PBS. **(D)** Quantitative analysis of Col1a1 and Col1a2 mRNA levels in Ja18$^{-/-}$

mice (n = 5) adoptively transferred with iNKT cells or PBS. **(E)** Experimental model of adoptive transfer iNKT cells. Quantitative analysis of IL-10 and IFN-γ mRNA levels from iNKT cells adoptively transferred into Ja18$^{-/-}$ mice (n = 5) fed MCS or MCD diet. Data represent mean relative expression ± SEM. *$P < 0.05$; **$P < 0.01$; ***$P < 0.001$ using two-tailed unpaired student's t-test.

Supplementary Video legends

Video S1. Intravital imaging of the migration behaviors of hepatic iNKT cells in untreated *Cxcr6*$^{Gfp/+}$ mice. The video was recorded for 10 min. Green, iNKT cells; Liver sinusoids (Red) labeled with Alexa Fluor 647-conjugated PECAM-1. Related to Figure 1.

Video S2. Clustering characteristics of iNKT cells after recruitment are shown in the livers of Cxcr6$^{Gfp/+}$ mice fed MCD diet for 3 days. The video was recorded for 20 min. Green, iNKT cells; Liver sinusoids (Red); labeled by Alexa Fluor 647-conjugated PECAM-1. Related to Figure 1.

Video S3. Behaviors of iNKT cells before and after activation by α-galactosylceramide (α-GalCer) in cell aggregation islands were compared in the same observation field. The video was recorded for 20 min. Green, iNKT cells. Related to Figure 2 .

Video S4. iNKT cells accumulated in areas with ruptured hepatocytes and iNKT cell aggregation sites were accompanied by enrichment in oxidative stress fragments. The video was recorded for 20 min. Green, iNKT cells; Red, DHE-ROS-594-stained oxidative stress debris; Blue, Hoechst33342-stained cell nuclei. Related to Figure 3.

Video S5. Three-dimensional reconstruction of Z-stack tile-merged confocal microscopy images of aggregated iNKT cells and oxidative stress debris in Cxcr6$^{Gfp/+}$ mice fed MCD diet for 3 days. Fifteen confocal z planes were recorded every 2 μm and reconstructed into a movie. Green, iNKT cell; Red, (DHE-ROS-594) fluorescence-labeled oxidative stress debris. The size of the image captured in the video is 50 μm × 50 μm × 30 μm. Related to Figure 3.

Video S6. Intravital imaging showing the dynamic interaction and clustering of Kupffer and iNKT cells in mice fed MCD diet for 3 days. The video was recorded for 30 min. Green, iNKT cells. Fuchsia, Kupffer cells stained with Alexa Fluor 647-conjugated F4/80 antibodies. Related to Figure 4.

Video S7. Three-dimensional reconstruction of Z stack tile-merged confocal microscopy images (patches of 2 focal plains) of aggregated iNKT cells and Kupffer cells in Cxcr6$^{Gfp/+}$ mice fed MCD diet for 3 days. Fifteen confocal z planes were recorded every 2 μm and reconstructed into a movie. The size of the image captured in the video is 50 μm × 50 μm × 30 μm. Green, iNKT cells; Fuchsia, Kupffer cells stained with Alexa Fluor 647-conjugated F4/80 antibodies. Related to Figure 4.

Video S8. Intravital imaging showing an iNKT cell approaching and encapsulating a free lipid droplet. The video was recorded for 15 min. Green, iNKT cells; Red, Nile red-labeled lipid droplets. Related to Figure 5.

Video S9. Three-dimensional reconstruction of Z stack tile images by 3D surface rendering showing interactions between aggregated iNKT cells and lipid droplets in an iNKT cluster in Cxcr6$^{Gfp/+}$ mice fed MCD diet for 3 days. Ten confocal z planes were recorded every 2.5 μm and reconstructed into a movie. Green, iNKT cells; Red, Nile red-labeled lipid droplets. The size of the image captured in the video is 40 μm × 40 μm × 25 μm. Related to Figure 5.

Video S10. Clustering characteristics of iNKT and Kupffer cells reappeared in Jα18$^{-/-}$ mice with adoptively transferred iNKT cells. Video 1 was recorded for 20 min. Green, iNKT cells; Red, DHE-ROS-594-stained oxidative stress debris; Video 2 was recorded for 20

min. Green, iNKT cells. Fuchsia, Kupffer cells stained with Alexa Fluor 647-conjugated F4/80 antibodies. Related to Figure 8.

Video S11. Three-dimensional reconstruction of Z stack tile-merged confocal microscopy images (patches of 2 focal plains). Compared with the adoptive transfer of PBS, liver fibrosis was reduced in Jα18$^{-/-}$ mice with adoptively transferred iNKT cells detected by second harmonic microscopic imaging. Fifteen confocal z planes were recorded every 2 μm and reconstructed into a movie. The size of the image captured in the video is 425μm × 425 μm × 30 μm. Red, Rhodamine fluorescent-labeled outline of hepatocytes; White, second harmonic signal-fibrosis imaging. Related to Figure 8.

Table S1: Primer Sequences

Gene name	Forward primer (5'->3')	Reverse primer (5'->3')
MMP8	CAGACAACCCCATCCAAC	AAGTAAATCTCCCCACGGA
MMP9	CACCGGCTAAACCACCTC	CGCCCGACACACAGTAAG
MMP12	CTGGGCTTCTCTGCATCT	GCTCCTGCCTCACATCA
MMP13	GGGGAGCCACAGATGAG	AACGCTCGCAGTGAAAAG
TIMP1	TCACTGTTTGTGGACGGA	AGGCTTCAGGTCATCGG
TIMP2	GAGAGGAAACCGAGCGA	GAGTTGCTGGCATGACG
TIMP3	ACACGGAAGCCTCTGAAA	CCTCTCCACAAAGTTGCAC
TIMP4	AGTGTCATGCCCTGGAGT	AGCCGAGTGGCAGAAGT
Apoa4	CACCTGAAGCCCTATGCC	CTCCTTGATCGTGGTCTGC
Dgat2	ACCCGACCCAGAAAGACA	TTCACCTCCAGCACCTCA
DpId1	ATCTGGTCATCAGCTCCCCT	CTTCCAGCAGCAACAACCAC
SmpdI3b	TGGCAATTGTGGAACGCTTG	AACATTCGGAAGCTGTCGGT
IL-6	ACAACCACGGCCTTCCCTAC	TCCACGATTTCCCAGAGAACA
TNF-α	ACGGCATGGATCTCAAAGAC	AGATAGCAAATCGGCTGACG
IL-1β	TGTCTTGGCCGAGGACTAAGG	TGGGCTGGACTGTTTCTAATGC
IL-5	ATGACTGTGCCTCTGTGCCTGG AGC	CTGTTTTTCCTGGAGTAAACTGG GG
IL-10	ACAGCCGGGAAGACA ATA AC	CAGCTGGTCCTTTGTTTGAAAG
IFN-γ	ACGGCACAGTCATTGAAAGCCTA GA	TGTCACCATCCTTTTGCCAGTTC C
COL1a1	CCCACCCCAGCCGCAAAGAG	GCCATGCGTCAGGAGGGCAG
COL1a2	CCGTTCCTTGACATTGCACCT	ACAACAGGTGTCAGGGTGTTA
GAPDH	TGCAGTGGCAAAGTGGAGATT	TCGCTCCTGGAAGATGGTGAT
